# Protein Quantitative Trait Loci Identify Novel Candidates Modulating Cellular Response to Chemotherapy

**DOI:** 10.1371/journal.pgen.1004192

**Published:** 2014-04-03

**Authors:** Amy L. Stark, Ronald J. Hause, Lidija K. Gorsic, Nirav N. Antao, Shan S. Wong, Sophie H. Chung, Daniel F. Gill, Hae K. Im, Jamie L. Myers, Kevin P. White, Richard Baker Jones, M. Eileen Dolan

**Affiliations:** 1 Department of Medicine, The University of Chicago, Chicago, Illinois, United States of America; 2 Committee on Genetics, Genomics and Systems Biology, The University of Chicago, Chicago, Illinois, United States of America; 3 Ben May Department for Cancer Research, The University of Chicago, Chicago, Illinois, United States of America; 4 Institute for Genomics and Systems Biology, The University of Chicago, Chicago, Illinois, United States of America; 5 Department of Health Studies, The University of Chicago, Chicago, Illinois, United States of America; 6 Department of Human Genetics, The University of Chicago, Chicago, Illinois, United States of America; 7 Committee on Clinical Pharmacology and Pharmacogenomics, The University of Chicago, Chicago, Illinois, United States of America; Georgia Institute of Technology, United States of America

## Abstract

Annotating and interpreting the results of genome-wide association studies (GWAS) remains challenging. Assigning function to genetic variants as expression quantitative trait loci is an expanding and useful approach, but focuses exclusively on mRNA rather than protein levels. Many variants remain without annotation. To address this problem, we measured the steady state abundance of 441 human signaling and transcription factor proteins from 68 Yoruba HapMap lymphoblastoid cell lines to identify novel relationships between inter-individual protein levels, genetic variants, and sensitivity to chemotherapeutic agents. Proteins were measured using micro-western and reverse phase protein arrays from three independent cell line thaws to permit mixed effect modeling of protein biological replicates. We observed enrichment of protein quantitative trait loci (pQTLs) for cellular sensitivity to two commonly used chemotherapeutics: cisplatin and paclitaxel. We functionally validated the target protein of a genome-wide significant trans-pQTL for its relevance in paclitaxel-induced apoptosis. GWAS overlap results of drug-induced apoptosis and cytotoxicity for paclitaxel and cisplatin revealed unique SNPs associated with the pharmacologic traits (at p<0.001). Interestingly, GWAS SNPs from various regions of the genome implicated the same target protein (p<0.0001) that correlated with drug induced cytotoxicity or apoptosis (p≤0.05). Two genes were functionally validated for association with drug response using siRNA: SMC1A with cisplatin response and ZNF569 with paclitaxel response. This work allows pharmacogenomic discovery to progress from the transcriptome to the proteome and offers potential for identification of new therapeutic targets. This approach, linking targeted proteomic data to variation in pharmacologic response, can be generalized to other studies evaluating genotype-phenotype relationships and provide insight into chemotherapeutic mechanisms.

## Author Summary

The central dogma of biology explains that DNA is transcribed to mRNA that is further translated into protein. Many genome-wide studies have implicated genetic variation that influences gene expression and that ultimately affect downstream complex traits including response to drugs. However, because of technical limitations, few studies have evaluated the contribution of genetic variation on protein expression and ensuing effects on downstream phenotypes. To overcome this challenge, we used a novel technology to simultaneously measure the baseline expression of 441 proteins in lymphoblastoid cell lines and compared them with publicly available genetic data. To further illustrate the utility of this approach, we compared protein-level measurements with chemotherapeutic induced apoptosis and cell-growth inhibition data. This study demonstrates the importance of using protein information to understand the functional consequences of genetic variants identified in genome-wide association studies. This protein data set will also have broad utility for understanding the relationship between other genome-wide studies of complex traits.

## Introduction

Pharmacogenomics aims to identify clinically actionable markers associated with response or toxicity; for oncology, evaluating genotype-phenotype relationships is particularly important because non-response and adverse events associated with chemotherapy can be life-threatening. Drug response and toxicity are thought to be multi-genic traits requiring whole genome studies to capture the most relevant variants. To complement clinical data and enhance discovery of genetic variants associated with sensitivity to drugs using a whole genome approach, we and others (reviewed by Wheeler and Dolan [1]) have developed cell-based models using International HapMap lymphoblastoid cell lines (LCLs). The genetic and expression environment for these cells has been well characterized thus allowing for genome-wide association studies (GWAS) and functional follow-up studies. Genetic variants associated with a given chemotherapeutic discovered in the LCL pharmacogenomic model have been replicated in clinical trials, arguably the most relevant system for biomedical science [2], [3], [4], [5], [6].

In addition to their value in pharmacogenomics discovery [7], [8], [9], [10], [11], LCLs have had broad utility as a discovery tool for genetic markers associated with many functional phenotypes, including: gene expression [12], [13], [14], [15], [16]; modified cytosines [17]; variation in mRNA decay rates across individuals [18]; DNase hypersensitivity [19]; and baseline micro RNA levels [20]. In addition, the LCL model has been used to identify genetic markers of inflammatory cell death [21], bipolar disorder [22], and response to serotonin reuptake inhibitors [23], [24]. Therefore, incorporating protein expression information into an existing dataset of genetic, epigenetic, mRNA expression, and drug sensitivity has the potential to identify novel candidates and mechanisms relevant to pharmacologic traits.

Previously, we reported that SNPs associated with inter-individual variation in cytotoxicity of chemotherapeutic agents in LCLs are enriched in expression quantitative trait loci (eQTLs) and separately, enrichment was observed for eQTLs associated with ten or more target genes [25]. SNPs that overlapped between preclinical LCL studies and outcomes of patients treated with the same drug were also enriched in eQTLs [2]. An implicit assumption in these analyses and studies of other complex traits is that mRNA transcript abundances are a suitable proxy measurement for their corresponding protein levels. However, recent data has demonstrated poor overall correlations between mRNA and protein expression [26], [27], [28], [29], [30].

To investigate the role of genomics in protein expression and the role protein expression plays in altering pharmacologic responses, we employed the micro-western array (MWA) [31], a method that is approximately 1000-fold more sensitive and has an ∼100-fold greater dynamic range than standard mass spectrometry methods and requires ∼200-fold less sample and antibody than standard immunoblotting methods [32], [33]. After screening 4,366 previously unvalidated antibodies targeting 1,848 transcription factors (TFs) and 200 well-validated antibodies targeting cell signaling proteins, we used MWAs and reverse phase protein arrays (RPPAs) to collect protein data regarding 441 protein isoforms from 68 HapMap Yoruba (YRI) LCLs. Baseline protein levels were evaluated for their correlations with cellular sensitivity to cisplatin and paclitaxel, two of the most widely-used and successful chemotherapeutics worldwide that are mechanistically distinct [34], [35], [36]. The measurement of proteins in HapMap LCLs is of great value to complement the extensive publicly available genetic information already available on these cell lines. Although LCLs are not tumor cells, upon transformation they are likely to have changes in pathways that control cell cycle and cell proliferation, which are relevant pathways for anti-cancer drugs. Furthermore, we identified genetic variants associated with chemotherapeutic sensitivity that acted through their effect on protein levels. We observed an enrichment of pQTLs in genome variants associated with pharmacologic phenotypes. We combined this information to identify proteins relevant for pharmacologic phenotypes through multiple independent SNPs throughout the genome.

## Results

### Biological replicates enable robustness in measurement of protein levels

**Figure 1:**
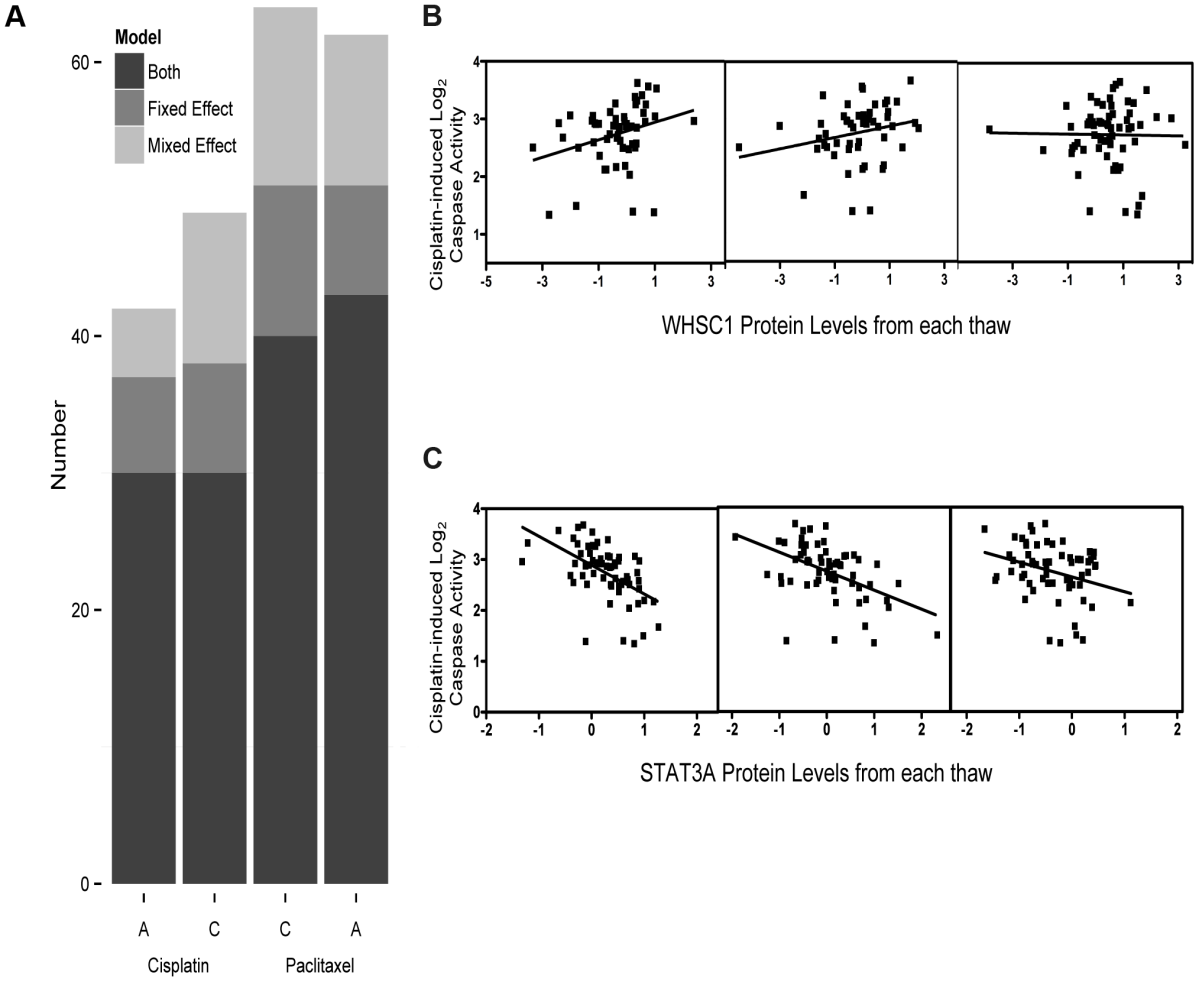
Protein levels regressed against four cytotoxicity phenotypes using fixed effect or mixed effect models representing the three biological replicates. We analyzed 441 protein levels against 5 µM cisplatin induced apoptosis and cytotoxicity and 12.5 nM paclitaxel apoptosis and cytotoxicity using both fixed effect and mixed effect modeling (a). The Y-axis represents the total number of protein-drug phenotype (A, apoptosis and C, cytotoxicity) correlations (p<0.05) using fixed effect (medium grey) or mixed effect (light grey) or those that showed a correlation for both methods (dark grey). Five micromolar cisplatin induced caspase activity correlated with WHSC1 protein levels demonstrates strong association (p = 0.009) using the fixed effect, whereas the individual thaw association reveals no association from the third thaw, resulting in a greater than p>0.05 MEM result (b). Five micromolar cisplatin-induced caspase activity correlated with STAT3A (∼90 kDa) protein levels across three thaws ranging had p<0.05 ranging from 0.02 to 1.6×10^−6^ and a mixed effect p-value of 1.55×10^−7^ (c).

Prior to our global analysis, a pilot study consisting of three independent biological replicates of six cell lines demonstrated significant variation not only among protein levels from different individuals, but also among cells thawed and propagated independently from the same individual. Based on a significant thaw effect explaining 3.75% of global protein expression variation (p = 0.01, *F* test), we measured baseline, steady-state protein levels from three independent thaws (thawed simultaneously) from each of 68 unrelated YRI LCLs to have a more accurate estimate of inter-individual variation in protein expression. These measurements were evaluated with both fixed effect (by averaging the three thaws) and mixed effect (by incorporating a random thaw effect per individual) models. Mixed effect modeling (MEM) allowed us to gain additional power from multiple measurements compared with simply averaging across the biological replicates in a linear model (Figure 1a). Relationships identified by fixed effect that had conflicting trends (i.e. positive and negative associations) across biological replicates were more likely to be false positives (Figure 1b) than the observations that were reproducible by MEM (across biological replicates) (Figure 1c); we therefore considered the MEM to be the more robust approach and used this method for all subsequent estimates of protein-drug associations.

### Relationship between drug phenotypes with protein levels

Cell growth inhibition and caspase 3/7 activation were measured following treatment of 68 unrelated YRI LCLs with cisplatin (5 µM) or paclitaxel (12.5 nM). Notably, the correlation between cytotoxicity and apoptosis was greater for paclitaxel (r*^2^* = 0.35) than cisplatin (r*^2^* = 0.04), indicating that apoptotic cell death was a larger contributor to paclitaxel-mediated cell growth inhibition compared with cisplatin (Figure S1). We also assessed the effect of date of cell thaw on cellular phenotypes and found a significant correlation across two independent thaws (Figure S2; p<0.0001 and r^2^>0.28 for cytotoxicity, p<0.003 and r^2^>0.38 for apoptosis).

From a starting pool of 4,366 antibodies, 198 antibodies producing a single predominant signal at the predicted molecular weight were carried forward for population-level quantification with the RPPA approach and 243 antibodies that displayed at least one band the size of the targeted protein isoform of interest with a signal-to-noise ratio ≥3 (but additional bands) were selected for subsequent population-level quantification by MWAs. We quantified the expression of 441 proteins across the same set of 68 individual LCLs for which we measured responses to chemotherapeutic agents. At an FDR of 20%, 64 proteins were associated with one or more of the four drug phenotypes. At p<0.05, 52 and 60 protein levels were associated with paclitaxel-induced apoptosis and cytotoxicity, respectively, and 47 and 39 proteins were associated with cisplatin-induced apoptosis and cytotoxicity, respectively. Table S2 details these nominal associations for each phenotype and Table 1 highlights the top three associations for each phenotype. We compared the overlap between the two drugs and identified four proteins that were unique to the apoptotic pathway including CDKN2B, PDK1, TFB1M and ZNF132. EP300 was the only protein exclusively associated with cytotoxicity for both drugs. This observation implies that loss of cell viability in response to these two drugs occurs through distinct mechanisms.

**Table 1:** Top proteins associated with cisplatin and paclitaxel phenotypes using a mixed effect model.

Protein	Gene Name	Phenotype	MEM p-value	Beta
p-S6.ribosomal.protein.(S240/244)	RPS6	Cisplatin Cytotoxicity	5.10E-04	1.08
NCKAP1L.75 kDa	NCKAP1L	Cisplatin Cytotoxicity	1.16E-03	−1.36
ZNF497	ZNF497	Cisplatin Cytotoxicity	2.56E-03	−0.84
**STAT3A (∼90 kDa)**	**STAT3**	**Cisplatin Apoptosis**	**1.46E-07**	**−0.78**
**STAT3B (∼80 kDa)**	**STAT3**	**Cisplatin Apoptosis**	**1.12E-04**	**−0.59**
**ENO1**	**ENO1**	**Cisplatin Apoptosis**	**3.16E-04**	**−0.48**
STAT3A (∼90 kDa)	STAT3	Paclitaxel Cytotoxicity	1.13E-05	1.23
GTF2F2	GTF2F2	Paclitaxel Cytotoxicity	3.10E-04	−1.15
ZNF266.75-100	ZNF266	Paclitaxel Cytotoxicity	3.12E-04	−0.89
**ENO1**	**ENO1**	**Paclitaxel Apoptosis**	**1.09E-05**	**−0.47**
**STAT3A (∼90 kDa)**	**STAT3**	**Paclitaxel Apoptosis**	**1.06E-03**	**−0.42**
**ZNF132**	**ZNF132**	**Paclitaxel Apoptosis**	**1.99E-03**	**−0.42**

**Figure 2:**
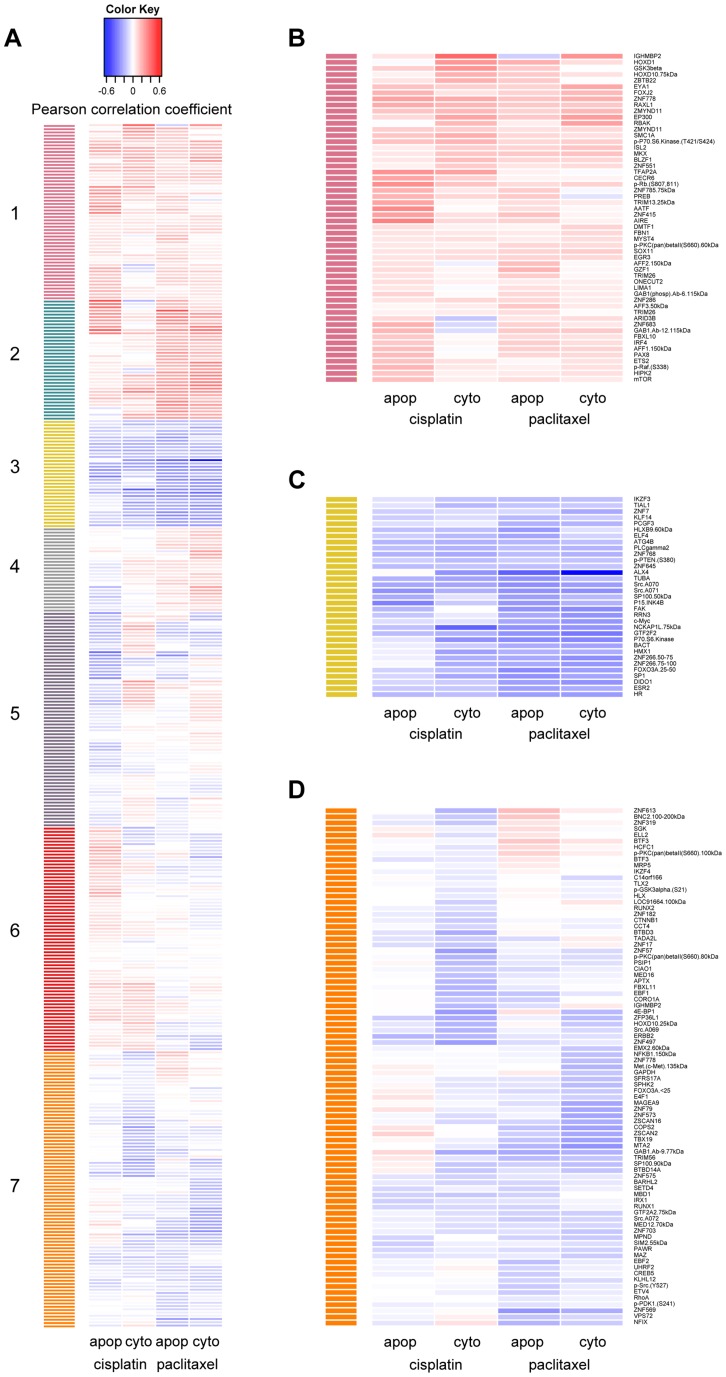
Establishing hierarchical clustering of baseline protein levels correlated with cisplatin and paclitaxel phenotypes. Hierarchical clustering was performed on 370 protein levels (rows) for 5 µM cisplatin apoptosis and cytotoxicity and 12.5 nM paclitaxel apoptosis and cytotoxicity (columns). The correlation for each protein-drug phenotype pair is indicated with blue showing increased protein levels associating with greater sensitivity to the drug, red showing increased protein levels associating with resistance to the drug and white indicating no correlation (a). The number of significant clusters was determined by performing 1000 permutations of the column correlations, clustering them, and selecting the number of observed clusters at a tree height that significantly exceeded all tree heights from the permutations (k = 7, p<0.001). Clusters 1 (b) and 3 (c) depict proteins that are related in the same direction to all cellular phenotypes. Cluster 7 (d) depicts proteins more related strongly to drug sensitivity through cytotoxicity than apoptosis.

Using hierarchical clustering of the drug-protein effect sizes, seven significant clusters were defined by permutation analysis (p<0.001) (Figure 2a). We were unable to identify any significantly enriched pathways due to the limited and biased background set of proteins evaluated; however, we did observe proteins of similar function within the clusters. Protein levels in cluster one (Figure 2b) were associated with increased resistance to both drugs when measured for either phenotype. Proteins in this cluster included many known metabolism-regulating proteins, DNA damage response factors, proteins associated with innate immune response, and transcription factors associated with various stages of developmental biology. Metabolism-regulating proteins included mTor, p70S6K(T421/S424), Gab1(Y627), GSK3beta, and ONECUT2. DNA damage-related proteins in cluster one included apoptosis antagonizing transcription factor (AATF) and structural maintenance of chromosomes protein 1A (SMC1A). Proteins with known associations to immune response included several ubiquitin ligases such as TRIM13 and TRIM26.

Protein levels in cluster 3 (Figure 2c) were associated with increased cellular sensitivity to both cisplatin and paclitaxel phenotypes and included many proteins related to calcium signaling: phospholipase C gamma 2 (PLCG2), c-Src (SRC) and focal adhesion kinase (FAK). Other proteins in cluster three included the tumor suppressor p15ink4b (CDKN2B), estrogen receptor beta (ESR2), beta actin (BACT), alpha tubulin (TUBA), and several transcription factors including c-MYC (MYC), Hairless homolog (HR), H6 family homeobox 1(HMX1), and ETS-related transcription factor Elf-4 (ELF4).

Protein levels in cluster 7 (Figure 2d) were associated more strongly with cellular sensitivity/resistance to drug cytotoxicity as compared with drug-induced apoptosis. Drug-induced cytotoxicity is a broad phenotype that includes cellular processes such as necrosis, cell death through apoptotic and non-apoptotic pathways, cell cycle arrest, and damaged cells undergoing DNA repair [37], whereas caspase 3/7 activation represents a specific process of cell death.

### Top protein quantitative trait locus implicates DIDO1 for paclitaxel-induced apoptosis

**Figure 3:**
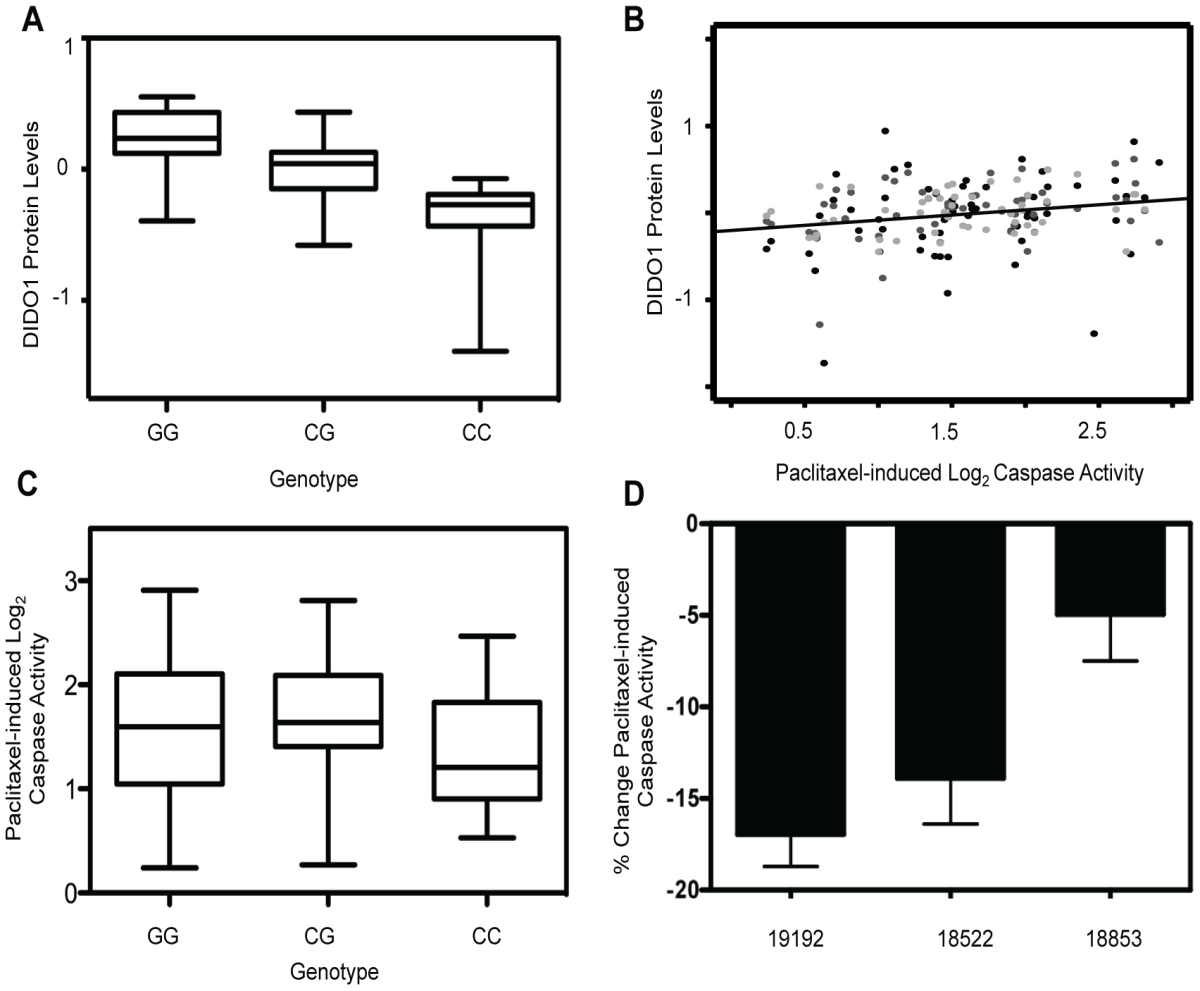
Identification of a protein quantitative trait locus relevant for paclitaxel-induced apoptosis. On chromosome 16, rs6834 genotypes were correlated with DIDO1 protein levels (p = 2.66×10^−15^) (a). DIDO1 protein levels were also significantly (p = 0.01) correlated with paclitaxel-induced apoptosis (b). The three shades of grey circles indicate data from each of the three thaws. Rs6834 was not significantly correlated with paclitaxel apoptosis (p>0.05); however, the CC individuals had both the lowest mean DIDO1 levels and lowest paclitaxel-induced apoptosis levels (c). Three LCLs were nucleofected with pooled DIDO1 or nontargeting control and apoptosis was measured 24 hrs after 12.5 nM paclitaxel (d). Mixed effect modeling revealed a significant (p = 0.005) reduction in caspase activity.

Upon evaluation of all proteins with a genome-wide significant pQTL, we identified one protein that was also associated with paclitaxel-induced apoptosis. The *trans* pQTL on chromosome 16, rs6834, was significantly correlated (p = 2.66×10^−15^) with death inducer-obliterator 1 (DIDO1) protein levels (Figure 3a). DIDO1 was in cluster 3 (Figure 2c), indicating that increased baseline levels conferred greater cellular sensitivity to both chemotherapeutic agents. DIDO1 protein levels were significantly correlated with paclitaxel-induced apoptosis (p = 0.01 r^2^ = 0.02; Figure 3b). However, the DIDO1 pQTL was not significantly associated with paclitaxel-induced apoptosis (p = 0.25, Figure 3c). Despite the lack of statistical significance (likely because of small sample size), the directionality was consistent with the observed protein relationship: cells containing two C alleles had lower levels of DIDO1 and lower paclitaxel-induced caspase 3/7 activation. DIDO1 mRNA levels were not associated with paclitaxel apoptosis (p>0.05), suggesting that this relationship was protein-specific.

Using RNA interference, we performed gene knockdowns in YRI LCLs and examined the effect of knockdown on paclitaxel-induced cytotoxicity and apoptosis. Three different LCLs were nucleofected with siRNA against DIDO1. Although knockdown levels varied considerably, the maximal degrees of protein knockdown observed for 24 or 48 hours in 18522, 18853, and 19192, were 20%, 48%, and 59%, respectively. When we pooled data from all cell lines and experiments using a MEM, knockdown of DIDO1 resulted in a significant (p = 0.005) decrease in paclitaxel-induced caspase activity. On average, paclitaxel-induced apoptosis was decreased by 11.9% in cells following knockdown of DIDO1 (Figure 3d).

### Enrichment of SNPs associated with chemotherapeutic phenotypes

**Figure 4:**
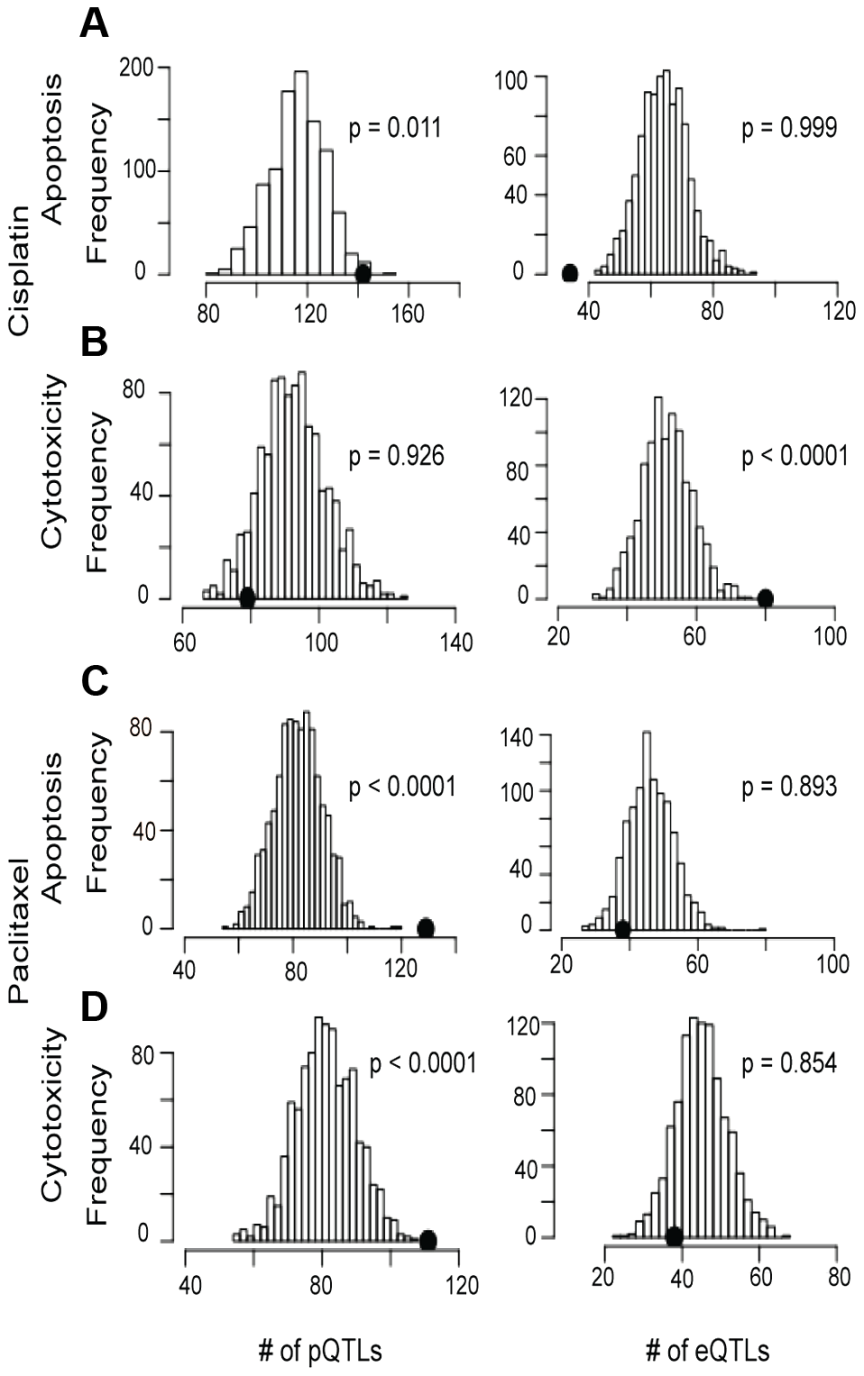
Evaluation of eQTL and pQTL enrichment associated with chemotherapeutic-induced phenotypes. Distribution of eQTL or pQTL count in 1000 permutations, each matching the MAF distribution of the chemotherapeutic drug sensitivity associated SNPs at p<10^−3^ and controlling for linkage disequilibrium using recombination blocks. The observed number of pQTLs (left plots) and eQTLs (right plots) are shown relative to these background permutations for cisplatin apoptosis (a) and cytotoxicity (b) and paclitaxel apoptosis (c) and cytotoxicity (d).

Using the pQTLs and eQTLs (unadjusted p<10^−4^) from the genes included in our protein dataset, we evaluated enrichment with paclitaxel and cisplatin-induced cytotoxicity and apoptosis associated SNPs at unadjusted p<10^−3^ (Figure 4). For cisplatin, only the apoptosis phenotype demonstrated pQTL enrichment (p<0.001) (Figure 4a, 4b, left panels). Conversely, both paclitaxel phenotypes demonstrated pQTL enrichment (Figure 4c, 4d). When evaluating eQTLs, only cisplatin cytotoxicity showed enrichment for eQTLs (Figure 4b). However, when evaluating all expressed genes, eQTLs showed enrichment for all drugs and phenotypes except for cisplatin-induced apoptosis (data not shown).

### Utilizing pQTLs to identify proteins implicated in cisplatin and paclitaxel cellular response

**Figure 5:**
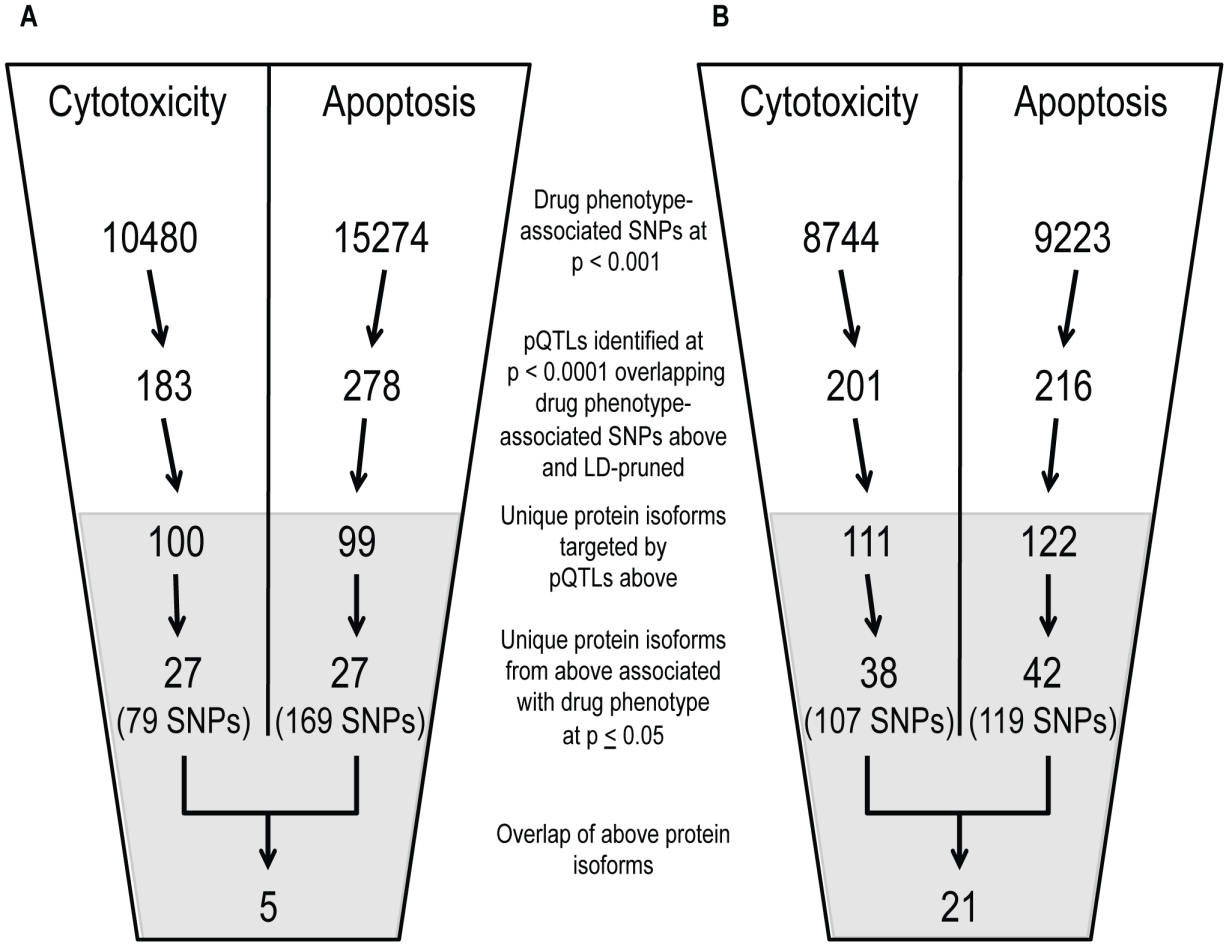
Identification of common proteins associated with differing phenotypes through independent pQTL signals for cisplatin and paclitaxel. Genome-wide association results (p<10^−3^) on two cellular phenotypes (growth inhibition and apoptosis) for both cisplatin (a) and paclitaxel (b) were analyzed for pQTLs. All SNP that were pQTLs (p<10^−4^) had their target proteins evaluated for correlation with the drug phenotype (p≤0.05). For each drug, the target protein overlap between cytotoxicity and apoptosis is indicated as the final number. The grey shading indicates the shift from numbers of variants to numbers of proteins and the SNPs presented in the grey area represent the number of SNPs targeting the proteins.

Using both cell growth inhibition and apoptosis as cellular phenotypes, we identified pQTLs (defined at p<10^−4^) associated with these phenotypes at p<0.001. From that overlap of pQTLs, we then analyzed the relationship between target protein levels and the respective drug phenotype (p≤0.05) (Figure 5). Overlapping GWAS signals identified five proteins for cisplatin phenotypes and 21 proteins for paclitaxel phenotypes (Table S3). For each phenotype, we also identified individual lists of proteins-pQTL pairs that both associate with cisplatin or paclitaxel phenotypes (Table S4). For cisplatin GWAS, there were 79 pQTLs targeting 27 proteins for cytotoxicity and 169 pQTLs targeting 27 proteins for apoptosis. For paclitaxel GWAS, there were 107 pQTLs targeting 38 proteins for cytotoxicity and 119 pQTLs targeting 42 proteins for apoptosis. Interestingly, the protein SRC was implicated through all four phenotypes.

**Figure 6:**
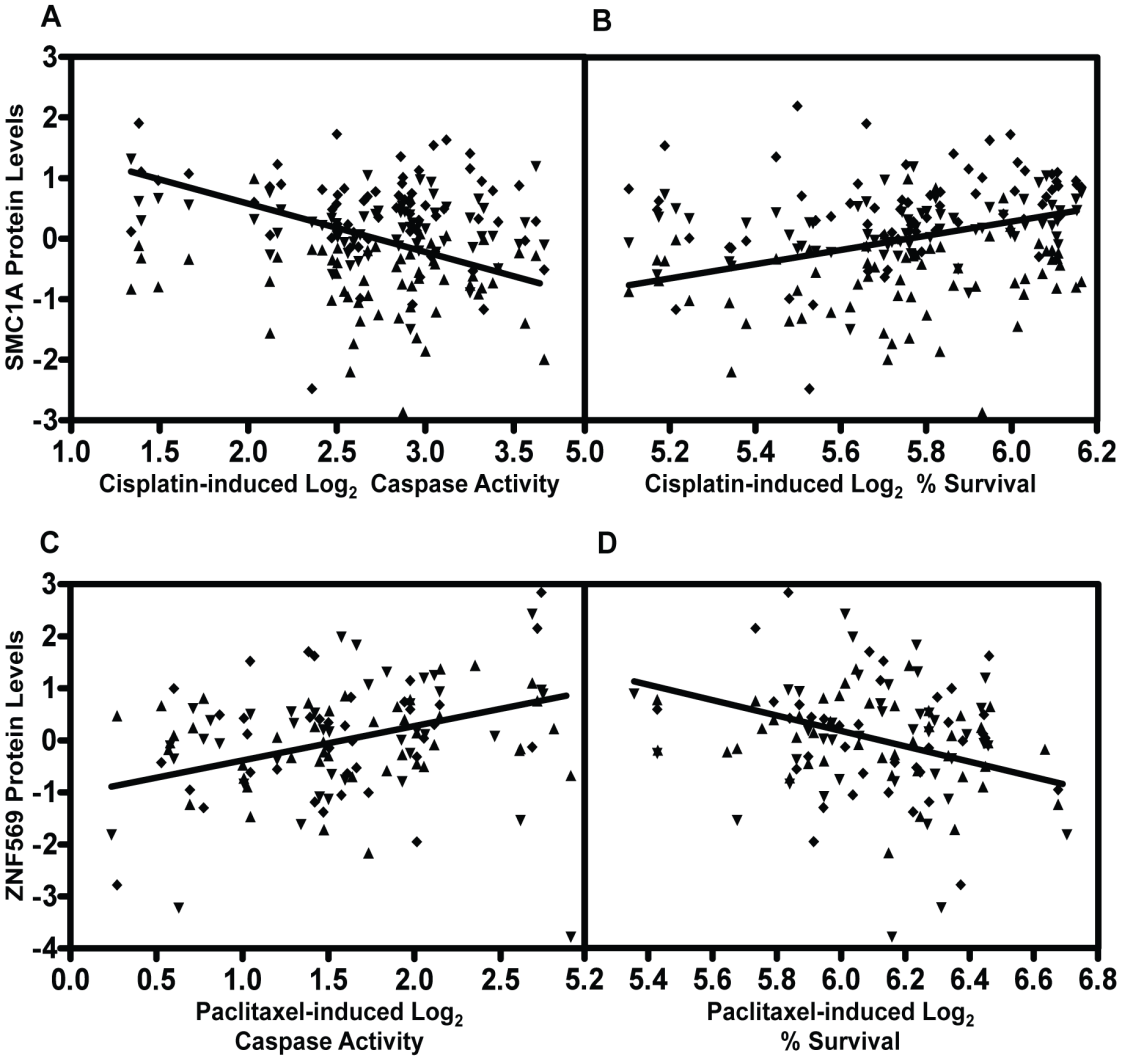
Genetic variants relevant in chemotherapeutic response implicated through protein effects. For proteins implicated through SNPs in both apoptosis and cytotoxicity GWASes, mixed effect modeling was performed to measure the direction of the relationships. SMC1A, structural maintenance of chromosomes 1A, was positively associated (p = 0.0007) with 5 µM cisplatin induced apoptosis (a) and negatively associated (p = 0.004) with 5 µM cisplatin-induced cytotoxicity (b). ZNF569, zinc finger protein 569, was negatively associated (p = 0.0002) with 12.5 nM paclitaxel-induced apoptosis (c) and positively associated (p = 0.0005) with 12.5 nM paclitaxel-induced cytotoxicity (d). All four plots display data from the three thaws represented by diamonds, triangles, or inverted triangles.

We prioritized proteins for functional studies using the apoptosis relationship for paclitaxel and the cytotoxicity relationship for cisplatin. Among the five proteins whose baseline expression levels associated with cisplatin cytotoxicity and apoptosis, we found structural maintenance of chromosomes 1A (SMC1A) to have the most significant relationship with cytotoxicity (p = 0.005, r^2^ = 0.039) (Figure 6a and 6b). We therefore selected it for further functional validation. SMC1A did not associate with either cisplatin phenotype at the mRNA level suggesting that this was a protein-specific relationship. Because more proteins were associated with paclitaxel-mediated apoptosis and cytotoxicity phenotypes, we prioritized functional follow-up based on a combination of p-values and q-values (to correct for multiple hypothesis testing). At p<0.005, five proteins were significantly associated with paclitaxel-induced apoptosis. Zinc finger protein 569 (ZNF569) (Figure 6c, 6d) had the lowest association q value. At the mRNA level, ZNF569 had a weak correlation with paclitaxel-induced apoptosis (p = 0.04, r^2^ = 0.06), but no relationship with paclitaxel-induced cytotoxicity. Table 2 lists the pQTLs that implicated SMC1A with the two cisplatin phenotypes and ZNF569 with the two paclitaxel phenotypes. We observed a different set of SNPs associated with each protein-drug pair that also associated with either apoptosis or cytotoxicity (Table 2).

**Table 2:** Protein QTLs implicating SMC1A for cisplatin and ZNF569 for paclitaxel.

Protein-Drug	SNP	MAF	Protein (p-value)	Apoptosis (p-value)	Cytotoxicity (p-value)	Most significant cis eQTL (gene-level RNA-Seq)	mRNA (p-value)
**SMC1A-cisplatin**	**chr13.44815900**	**0.12**	**5.36E-05**	**NA**	**4.93E-05**	**SLC25A30**	**0.041**
	**chr15.30332097**	**0.11**	**8.85E-06**	**NA**	**8.81E-04**	**FMN1**	**0.016**
	**rs17159458**	**0.20**	**2.14E-05**	**NA**	**6.61E-04**	**C7orf67**	**5.33E-03**
	**rs10423794**	**0.16**	**4.46E-05**	**NA**	**7.25E-04**	**ZNF578**	**0.030**
	**rs905495**	**0.22**	**2.49E-05**	**NA**	**1.23E-04**	**NA**	**NA**
	**chr12.114791277**	**0.06**	**2.84E-05**	**9.07E-06**	**NA**	**NA**	**NA**
	**chr19.24361339**	**0.23**	**2.2E-05**	**6.10E-04**	**NA**	**ZNF726**	**9.34E-03**
	**chr4.65471656**	**0.10**	**4.23E-05**	**6.29E-04**	**NA**	**NA**	**NA**
	**chr8.134151684**	**0.38**	**9.77E-05**	**6.05E-04**	**NA**	**NA**	**NA**
	**rs2256292**	**0.25**	**7.74E-05**	**3.19E-04**	**NA**	**NA**	**NA**
	**rs7186500**	**0.26**	**9.56E-05**	**4.00E-05**	**NA**	**NA**	**NA**
ZNF569- paclitaxel	chr1.68459252	0.19	4.15E-05	5.11E-04	NA	RP11-518D3.4	0.011
	chr12.118319993	0.49	3.04E-06	8.15E-04	NA	NME2P1	0.030
	chr2.indel83558053	0.22	6.77E-05	7.10E-04	NA	NA	NA
	rs6958145	0.32	5.03E-05	4.01E-04	NA	NA	NA
	rs9486877	0.29	1.33E-05	2.87E-04	NA	SESN1	0.012
	chr1.98729516	0.06	1.7E-05	NA	8.14E-04	NA	NA

Protein QTLs that also associate with either apoptosis or cytotoxicity are shown for each protein with bold indicating SMC1A and cisplatin and non-bold indicating ZNF569 and paclitaxel relationships. NA indicates that the p-value was greater than 0.001 for the drug phenotypes and 0.05 for the cis-eQTL associations.

**Figure 7:**
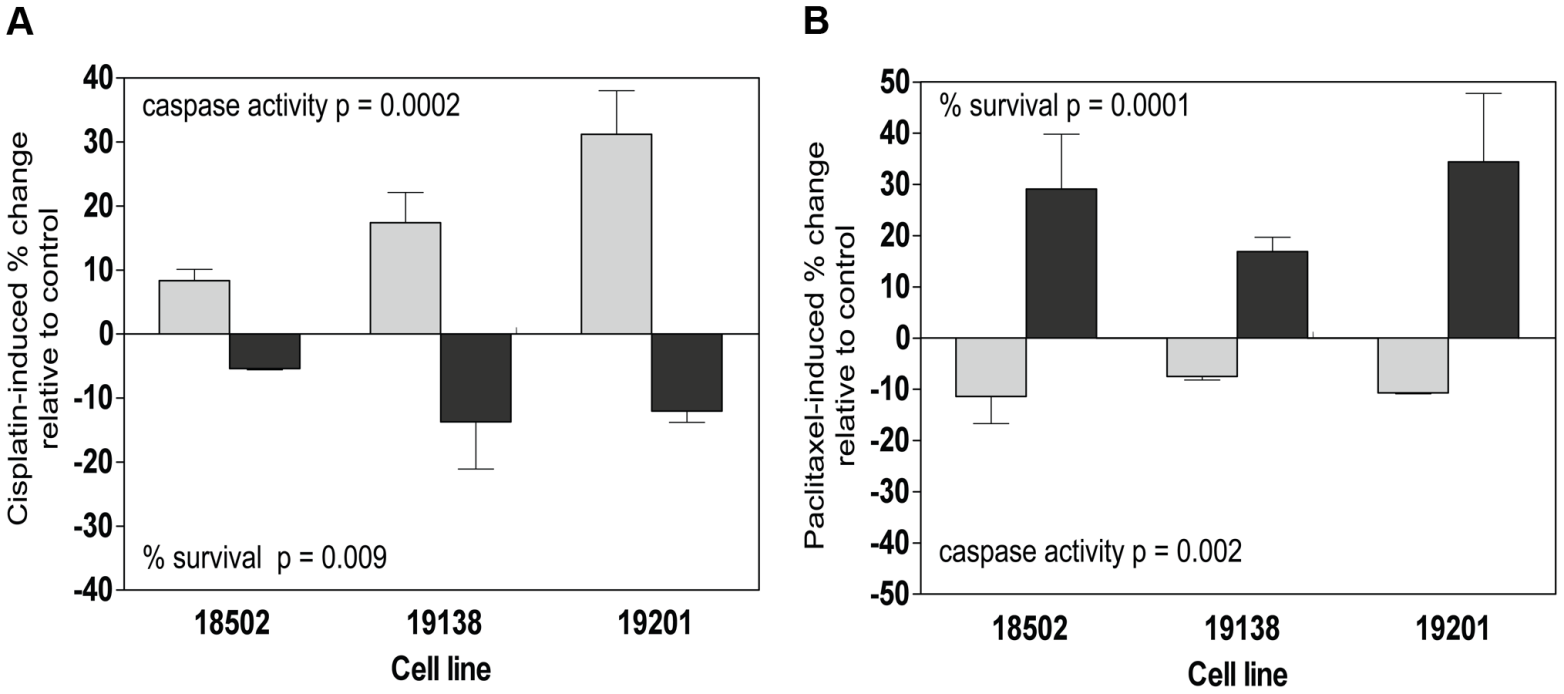
Functional validation of SMC1A and ZNF569. Three lymphoblastoid cell lines (18502, 19138, 19201) were evaluated 24 h and 48 h following 5 µM cisplatin treatment for cytotoxicity and apoptosis following nucleofection of pooled SMC1A and non-targeting control (a). Three lymphoblastoid cell lines (18502, 19138, 19201) were evaluated 24 h and 48 h following 12.5 nM paclitaxel treatment for cytotoxicity and apoptosis following nucleofection of pooled ZNF569 and non-targeting control (b).

Because independent pQTLs associated with the drug-induced phenotypes, we functionally validated the relationship of these proteins with their respective drug-induced phenotypes. We selected three LCLs (18502, 19138,19201) with mid to high protein expression and performed siRNA nucleofection. We assessed knockdown at 24 and 48 hours post nucleofection. Knockdown of SMC1A protein levels varied across the cell lines; we did not observe more than 57%, 71%, and 62% protein knockdown for 18502, 19138, and 19201, respectively, for either time point. Using a MEM to examine the effect across cell lines, we determined that knockdown of SMC1A resulted in a 19% increase in apoptosis (p = 0.0002) and a 10.4% decrease in cell survival (p = 0.009) in response to cisplatin (Figure 7a). Knockdown of ZNF569 protein levels varied across the cell lines, but we observed no more than 45%, 58%, and 54% protein knockdown across 18502, 19138, and 19201, respectively, for either time point. Using a MEM to combine the effect across cell lines, knockdown of ZNF569 resulted in a 9.9% average reduction in apoptosis (p = 0.002) and a 26.8% increase in cell growth inhibition (p = 0.0001) (Figure 7b) in response to paclitaxel.

### Role of growth in protein-drug relationships

Because growth rate has been previously identified as a heritable trait that is relevant in pharmacologic studies, we evaluated the relationship between steady state protein levels and intrinsic growth rate [38] for the proteins measured. Approximately 10% (45/441) of the proteins were correlated with growth at p<0.05 (Table 3). Notably, SMC1A protein levels were significantly correlated with growth rate (p = 0.0007), whereas ZNF569 protein levels were not (p>0.05) (Figure S3). When we adjusted for growth rate, the association of SMC1A protein levels with cisplatin phenotypes was no longer significant (p>0.05).

**Table 3:** Proteins associated with growth.

Protein	Gene	P-Value	Rho
p-S6.ribosomal.protein.(S240/244)	RPS6	2.58E-05	−0.492
NCKAP1L.75 kDa	NCKAP1L	6.66E-04	0.408
SMC1A	SMC1A	6.72E-04	−0.405
IRF5	IRF5	6.86E-04	−0.405
Src.A070	SRC	1.33E-03	0.384
Src.A071	SRC	1.86E-03	0.373
TAF15	TAF15	2.56E-03	−0.365
c-Myc	MYC	4.57E-03	0.341
ATG4B	ATG4B	5.38E-03	0.346
ZNF266.50-75	ZNF266	5.84E-03	0.332
ZNF778	ZNF778	6.31E-03	−0.356
HMX1	HMX1	8.07E-03	0.320
STAT3A (∼90 kDa)	STAT3	8.39E-03	−0.318
PKCzeta(C24)	PRKCZ	1.00E-02	−0.311
p-PTEN.(S380)	PTEN	1.02E-02	0.311
TULP1	TULP1	1.13E-02	0.306
GTF2F2	GTF2F2	1.15E-02	0.306
TUB	TUB	1.28E-02	0.301
RhoC	RHOC	1.33E-02	−0.309
ZBTB22	ZBTB22	1.42E-02	−0.297
DIDO1	DIDO1	1.51E-02	0.294
STAT3B (∼80 kDa)	STAT3	1.54E-02	−0.294
ZMYND11	ZMYND11	1.65E-02	−0.290
BACT	ACTB	1.79E-02	0.287
AFF2.50 kDa	AFF2	2.23E-02	−0.279
MYB	MYB	2.28E-02	−0.285
PLCgamma2	PLCG2	2.35E-02	0.275
p65	RELA	2.53E-02	−0.305
ENO1	ENO1	2.53E-02	−0.272
IRF5	IRF5	2.58E-02	−0.271
RRN3	RRN3	2.58E-02	0.326
TUBA	TUB	2.66E-02	0.303
HIVEP1	HIVEP1	2.79E-02	−0.269
SPHK2	SPHK2	2.91E-02	0.278
FOXO4	FOXO4	3.14E-02	−0.262
KLHL14	KLHL14	3.36E-02	−0.372
TBX19	TBX19	3.72E-02	0.254
TIAL1	TIAL1	3.84E-02	0.260
MED12.70 kDa	MED12	3.95E-02	0.253
FAK	PTK2	3.96E-02	0.251
HOXC6	HOXC6	4.10E-02	−0.249
FBXL10	KDM2B	4.42E-02	−0.249
FBN1	FBN1	4.53E-02	−0.244
IRF4	IRF4	4.54E-02	0.244
EP300	EP300	4.97E-02	−0.239

At p<0.05, approximately 10% of the 441 proteins assayed correlate with cell growth including SMC1A.

## Discussion

In this study, we evaluated 4,366 antibodies targeting 2,048 unique proteins. From this set, we identified antibodies targeting 441 protein isoforms expressed at baseline in LCLs and quantified them across three biological samples from 68 YRI LCLs. The use of multiple biological samples allowed us to implement mixed effects modeling to increase the robustness of our observations. Many protein expression levels were correlated with sensitivity to two cellular phenotypes (cytotoxicity and apoptosis) of two chemotherapeutic agents: paclitaxel and cisplatin. We validated one such finding through knockdown of DIDO1 in three LCLs, which resulted in a decrease in paclitaxel-induced apoptosis. Quantitative trait loci for pharmacologic phenotypes were compared to quantitative trait loci for protein expression to better understand the functional significance of genetic variants contributing to inter-individual variability in drug response. For each drug, we identified overlapping and unique sets of genetic variants associated with protein expression that were also correlated with drug-induced apoptosis and cytotoxicity. We further validated two such proteins through gene knockdown and concomitant modulation of cellular sensitivity to drug treatment: SMC1A levels were associated with resistance to cisplatin treatment, and ZNF569 levels were associated with sensitivity to paclitaxel treatment.

This study illustrates the utility of applying a highly-sensitive, novel, antibody-based technology to simultaneously measure many proteins across a large set of individuals. Using this method, we identified hundreds of novel genome loci that uniquely influence the expression of proteins that ultimately influence the sensitivity of cells to chemotherapeutic agents through both caspase 3/7 activation and other pathways leading to loss of cell viability. We evaluated protein expression in the International HapMap LCLs because these samples have previously been used for many studies relating genetics to gene expression [14], [16], [39] and cellular phenotypes [1], thus allowing us to perform comprehensive studies of genetics, protein expression, and pharmacology. LCLs are immortalized B-lymphocytes and, as a result, represent “non-cancerous” cells that may provide us with important protein targets for ameliorating bone marrow suppression. However they also have some of the pathways relevant to anti-cancer drugs. We specifically chose the YRI population because of their greater genetic diversity relative to other populations. We expect that this data will have wide applicability to other genetic and pharmacological studies because of the important addition of protein levels to other studies.

Whereas polymorphisms in coding regions that affect amino acid composition would seem to have the greatest effect on drug response, genetic variation that affects transcript abundance level has also been shown to affect drug response [25]. A disproportionate number of drug response associated SNPs in a broad array of chemotherapeutic agents are eQTLs and are associated with the transcriptional expression level of multiple genes [25]. However, our work has demonstrated poor global correlations between inter-individual mRNA and protein levels (unpublished data). Therefore, functional annotation of pharmacologic SNPs and their relationships with proteins may result in important new discoveries as it has in this study. We note that 46,863 of the 121,484 trans pQTLs identified at P<10^−4^ are also cis-acting eQTLs (within 1 Mb upstream of the transcription start state to 1 Mb downstream of the transcription end site) for at least one of the 18,227 gene models quantified by RNA-Seq at P<0.05. This proportion (38.6%) is statistically enriched compared with the proportion of all single nucleotide variants genome-wide that are cis-eQTLs (36.6%, Fisher's exact test P<2.2×10^−16^, odds ratio = 1.09), suggesting that cis-acting may contribute to some extent to underlying trans-genetic regulation of protein levels.

Because we performed multiple analyses to examine overlap and enrichment of protein and drug QTL, the p-value thresholds used in this study were more permissive relative to that typically used for genome-wide analyses. By contrast to various chemotherapeutics that exhibit GWAS enrichment in eQTLs [25], paclitaxel GWAS results were not enriched in eQTLs; however, we identified enrichment in pQTLs for both paclitaxel-induced apoptosis and cytotoxicity phenotypes. Therefore, genetic variants associated with the level of a protein appear to be more important for sensitivity to this drug than mRNA regulatory variants. We functionally validated one of these observations, DIDO1, by siRNA knockdown.

DIDO1 is a tyrosine phosphorylated transcription factor that is localized to the nucleus [40]. DIDO1 was also found within cluster 3, which contained proteins with increased baseline levels correlating with greater cytotoxicity and apoptosis to each chemotherapeutic agent tested. DIDO1 is generally believed to function through apoptosis-related processes; however, it has also been suggested to function in mitotic division based on gene overexpression in mice [41]. This proposed function provides a clear mechanistic connection to paclitaxel, a drug that kills cells through microtubule inhibition.

Both paclitaxel and cisplatin have been in use for decades, and significant effort has been expended to identify strategies that result in increased tumor sensitivity to these agents, including targeting the activity of drug resistance pathways. However, this approach is only successful if the cancerous and non-cancerous cells differ in their response to modulation. Improving the therapeutic index for patients occurs if the “modulating agent” increases the sensitivity of chemotherapy in the tumor while decreasing toxicity in non-tumor tissues. This study offers an opportunity to identify the relationship between transcription factors and signaling molecules and drug sensitivities in a non-tumor environment. For example, high levels of proteins identified in cluster 3 were associated with greater sensitivity to both cisplatin and paclitaxel; yet several of these proteins including c-Src [42], [43] and c-Myc [44], [45] have been shown to be overexpressed in tumor cells and their expression correlates with paclitaxel or cisplatin resistance. c-Src tyrosine kinase is overexpressed in a high proportion of ovarian cancers and ovarian cancer cell lines. Its inhibition, either pharmacologically or through gene knockdown, results in an increase in sensitivity of ovarian cancer cells to paclitaxel and cisplatin [43]. The increased cytotoxicity in response to c-Src inhibition was associated with a large increase in processing and activation of caspase-3. Our data support these proteins as potential drug targets, because reducing their levels in LCLs would result in lower sensitivity to the toxic effects of cisplatin and paclitaxel in contrast to cancerous cells. We anticipate that this dataset will therefore have great utility for the development of novel modulators of chemotherapy.

Although LCLs are a more likely model for toxicity, we identified several relationships that have been recapitulated in tumor response. Signal transducer and activator of transcription 3 (STAT3) had the strongest negative associations with cisplatin- and paclitaxel-induced apoptosis, suggesting high levels of STAT3 protein conveyed drug resistance. STAT3 mRNA expression has previously been reported to be associated with cisplatin resistance in many cancer types, including head and neck [46], small cell lung carcinoma [47], and human epidermoid cancer cells [48], in which the CRE/ATF binding elements in the STAT3 promoter were shown to be important for mediating cisplatin resistance. STAT3 mRNA expression has also been implicated in paclitaxel resistance. Knockdown of STAT3 conveyed sensitivity to paclitaxel in lung cancer cell lines [49]. STAT3 has been hypothesized as a potential target to modulate paclitaxel sensitivity in cancer patients [50]. PTEN is also an example of same direction of effect in LCLs and cancer cells, however unlike STAT3, increased levels of PTEN convey sensitivity. Recent studies have demonstrated that PTEN has the ability to enhance cancer cell sensitivity to particular anticancer agents. PTEN might reverse the chemoresistance of human ovarian cancer cells to cisplatin through inactivation of the PI3K/AKT cell survival pathway and may serve as a potential molecular target for the treatment of chemoresistant ovarian cancer [51].

SMC1A is part of the multi-protein cohesion complex required for sister chromatid cohesion. This cohesion complex has been shown to interact with the BRCA1 DNA repair protein and has been shown to be phosphorylated by ATM, a serine/threonine kinase activated by DNA double-strand breaks [52]. The cohesion complex has also been shown to be important for expression regulation and genomic stability [53]. Mutations in SMC1A have been shown to cause Cornelia de Lange syndrome, a multisystem developmental disorder with defects ranging from limb formations to cardiac, gastrointestinal, growth and cognitive systems [53]. Coding variants have also been identified in colon cancer [54] and have been implicated in impairing cellular response to toxic treatment [55]. Accumulated SMC1A protein has been linked to bortezomib-induced cell death, demonstrating its relevance for another chemotherapeutic agent [56], but this is the first study implicating SMC1A for cisplatin-induced cellular response. Recently, Wip1, an important signaling protein in cellular growth following DNA damage, has been identified as an upstream regulator of SMC1A [57], further suggesting an important role for this protein in cancer and chemotherapeutic response. SMC1A has also been linked to cellular growth rate and was identified within cluster one which included proteins whose levels were associated with reduced cytotoxicity and apoptosis phenotypes across both drugs.

Another protein we functionally validated associated with paclitaxel, ZNF569, was a notable candidate because it has been functionally implicated as a transcriptional repressor that suppresses MAPK signaling [58]. Because of the importance of MAPK signaling in breast cancer [59] and the common use of paclitaxel as a breast cancer therapy [60], this association presents an interesting biological mechanism and potential therapeutic marker. ZNF569 is supported in our data as a transcriptional suppressor of MAPK signaling, because lower ZNF569 protein levels were correlated with increased cellular survival. In addition, ZNF569 was also found in the cluster of proteins that negatively correlated more strongly with cytotoxicity than apoptosis for both drugs, perhaps indicating a role for ZNF569 in cell growth inhibition unrelated to caspase 3/7 activation.

Notably, this study focused on two widely used but mechanistically distinct agents. By examining two distinct cell phenotypes, cell growth inhibition and caspase 3/7 activation, our study identified proteins associated with different cell signaling pathways responsible for cell growth inhibition. Although our study did not reveal candidates with strikingly high effect sizes that were predictive of drug sensitivity, it revealed many unique proteins whose expression levels were correlated with phenotypic measurements for a single drug. This observation is consistent with multiple proteins contributing small influences to drug sensitivity. The protein data collected in this study allowed us to gain a new understanding of the potential mechanisms and pathways relevant for cell viability and the genetic variants regulating those proteins.

Interpreting GWAS results continues to present challenges; increasingly, eQTL studies are being used to inform [25], [61], [62] interpretation of these results and are the focus of expanded studies to understand biological mechanisms [63], [64]. These association tests have been extended to other functional units in the genome from microRNAs [20] to DNA hypersensitivity sites [19] and modified cytosines [17]. The main factor limiting the inclusion of proteins in GWAS studies has been the lack of a reliable, high-throughput methodology to quantify them across populations of individuals. The approach described in this study, including the newly developed microwestern array [32], has started to bridge that technological gap [33], and this study demonstrates the utility of targeted protein-omic datasets to understand cellular phenotypes and genomic studies.

## Materials and Methods

### Cell lines

YRI LCLs derived from unrelated individuals from the population residing in Ibadan, Nigeria (n = 68) were chosen for consistency with publicly available mRNA expression data on a single population [16]. LCLs were cultured in RPMI 1640 media containing 20 mM L-glutamine and either 15% fetal bovine serum (Hyclone, Logan, UT) for baseline protein quantification, cisplatin and paclitaxel apoptosis and cisplatin cytotoxicity experiments or bovine growth serum (Hyclone, Logan, UT) for paclitaxel cytotoxicity experiments. Cell lines were diluted three times per week at a concentration of 300,000–350,000 cells/mL and maintained in a 37°C, 5% CO_2_ humidified incubator. Medium and components were purchased from Cellgro (Herndon, VA).

### Drug-induced cell apoptosis and cytotoxicity phenotypes

Drug-induced apoptosis and cytotoxicity phenotypes were determined at 5 µM cisplatin and 12.5 nM paclitaxel. Both drugs were prepared as described previously: cisplatin [65] and paclitaxel [66]. The cytotoxic effect of cisplatin [65] and paclitaxel [66] was determined using a short-term cellular growth inhibition assay, and the apoptotic effect was measured using a caspase 3/7 activity detection reagent Caspase-Glo 3/7 (Promega Corporation, Madison, WI).

### Protein isolation

Three independent thaws constituting biological replicates of 68 unrelated YRI cell lines were propagated and pelleted (5.1 million cells per pellet). Cells were spun at 400 RPM, aspirated, and washed in ice-cold PBS. This process was repeated twice and then the pellets snap frozen in liquid nitrogen and placed at −80 degrees. Total protein was extracted by re-suspension in 1.0 mL of 1.5% SDS lysis buffer (240 mM Tris-acetate, 1.5% w/v SDS, 0.5% w/v glycerol, 5 mM EDTA) containing 50 mM DTT, protease inhibitors (1 µg/mL aprotinin, 1 µg/mL leupeptin, 1 µg/mL pepstatin), and phosphatase inhibitors (1 mM sodium orthovanadate, 10 mM β-glycerophosphate). To ensure complete protein denaturation, samples were boiled for 10 min, sonicated for 10 min (alternating 30 s on, 30 s off) with a Bioruptor (Diagenode), and concentrated to 5–10 µg/µL using a 96-well micro-concentration device with a 10 kDa molecular weight cutoff (Millipore).

### Pilot study

To identify sources of steady-state protein expression variation, we performed a pilot study to quantify 21 proteins across three independent cultures from two independent thaws from two YRI LCLs (NA18861 and NA19193). We performed a multifactorial ANOVA to assess the proportion of protein expression variation explained for each of these variables across all proteins. We observed a significant thaw effect explaining 3.75% of global protein expression variation (p = 0.01, *F* test), whereas culture only explained 0.13% of protein expression variation (p = 0.85, *F* test). Using a mixed-effects model with a random nested effect, (1|individual/thaw/culture), only 2.71×10^−14^% of protein expression variation was between cultures within thaws, whereas 5.29% of variation was between thaws within individuals.

### Protein quantification and analysis with pharmacologic phenotypes

Initially, three biological replicates for each of 11–12 individuals were pooled together into six pools for screening 4,366 rabbit polyclonal antibodies at a 1∶1000 dilution. Printing, gel fabrication, horizontal semidry electrophoresis, transfer, blotting, and scanning were performed as in Ciaccio *et al.* [32], permitting 96 antibodies to be screened over six pooled lysates per MWA. The 4,366 antibodies were directed against 1,848 unique TFs and 200 unique cell signaling proteins. Of this set, 198 antibodies producing a single predominant band the size of the targeted protein isoform of interest with a signal-to-noise ratio ≥3 were selected for subsequent population-level quantification by RPPAs; antibodies that displayed at least one band the size of the targeted protein isoform of interest with a signal to noise ratio ≥3 but additional bands were selected for subsequent population-level quantification by MWAs. This approach ultimately allowed us to quantify protein levels from 441 antibodies (341 TF and 100 signaling) directed at 391 unique protein isoforms across three biological replicates of 68 LCLs. Additional antibody details are listed in Table S5.

For RPPA quantification, four technical replicates of each of three biological replicates of all 68 individuals were spotted using a noncontact piezoelectric microarrayer (GeSiM Nanoplotter 2.1E) onto nitrocellulose membranes (Biorad). Serial dilutions of each of the six pooled lysates used for the original antibody screen and an A431 skin carcinoma cell line control were also printed for each array to ensure the linearity and quality of the antibody signal. Features with background-subtracted integrated intensities <0 or signal to noise ratio <3 (*Z* test p>0.05) were identified in each array and excluded from further analysis. The distributions of background-corrected integrated intensities for all features on each array were first log_2_-quantile normalized using the limma package in R to correct for overall antibody hybridization efficiency differences in the signal. The relative expression of a given protein for a sample was then quantified using the linear model (1) 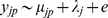, where *μ_jp_* is the log-quantile normalized, background-corrected integrated intensity of sample *j* on array *p*, λ*_j_* is the effect due to sample *j* across all arrays in a print (due to differing amounts of total protein spotted on the array for each sample), estimated by median*_j_*(*c_jp_*). Odyssey output text files were parsed in Python and quantified and normalized in R.

For micro-western quantification, three technical replicates of each of the three biological replicates of all 68 individuals were spotted as above onto polyacrylamide gels. Gel fabrication, horizontal semidry electrophoresis, transfer, and scanning were performed as in Ciaccio *et al.* [32] with the exception of separating each blot into four quadrants rather than using a 96-well gasket to permit 68×3 = 204 samples to be quantified with a single antibody on a single quadrant. Feature extraction and data normalization were performed as with RPPAs. For antibodies that produced multiple bands (signal to noise ratio >3), all isoforms were quantified and their relative molecular weights recorded. The expression of a given protein for an individual was quantified using the above linear model (equation 1) with the addition of a batch term (*β*) to correct for global intensity distribution differences across multiple microwesterns for the same antibody. For replicates within platforms for the same antibody across the entire population, we took the average of the expression measurements. For replicates across platforms, we selected the platform that yielded the highest median background-corrected integrated intensity. To reduce the inflated effect of technical noise because of low antibody signals and provide more accurate inter-individual protein expression measurements, antibodies in the bottom deciles of median background-corrected integrated intensities or in the top deciles of technical CVs for either platform were flagged and eliminated from further comparative analyses.

For each protein measurement from either method, we constructed linear mixed effects models 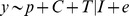, in which *p* is the array- and sample-load normalized integrated signal intensity for all biological replicates of all individuals comprising the population, *C* is the fixed effect of the drug, *T*|*I* is the random thaw effect per individual, and *e* is the residual error. The model was fitted to each protein by residual maximum likelihood using the lmer function in the R package lme4 (v 0.999999-0). This mixed effect model incorporates the direction of effect for each biological replicate and insures that those with conflicting directions would result in a less significant p-value. Fixed effect p-values for covariates were estimated using the pamer.fnc function in the LMERConvenienceFunctions package (v 1.6.8.3). The significance of covariate effects was assessed by estimating false discovery rates using Storey's q-value method.

*Hierarchical Clustering.* Hierarchical clustering was performed in R using Euclidean distance and the Ward method in hclust() for the standardized coefficients between the regression of 370 protein isoforms (rows) by the four drug phenotypes (columns), with the apoptosis coefficients inverted to match directionality with the cytotoxicity coefficients. The number of significant clusters was determined by performing 1000 permutations of the column coefficients, clustering them, and selecting the number of observed clusters at a tree height that significantly exceeded all tree heights from the permutations (k = 7, p<0.001).

### Genome-wide association studies

HapMap genotypes were obtained from the 1000 genomes, June 2011, phase I, low-pass whole genome SNP genotype release (www.1000genomes.org). Missing values were imputed by BIMBAM (v 1.0) using the default parameters to derive mean imputed genotypes. SNPs with MAF<0.05 and SNPs with significant deviation from Hardy-Weinberg equilibrium (Fischer's exact test, p<0.001) were excluded, reducing the set to 9,345,571 SNPs and indels for association analyses. To ensure that low MAF SNPs were not generating spurious associations due to outliers, we compared the MAF distribution of SNPs associated with protein and drug phenotypes with all SNPs (Figure S4). The average MAFs for protein (.17) and drug (.15) associations do not show a bias as compared with the genome (.16). Each protein expression measurement was inverse normal transformed prior to association analysis. Drug-induced cytotoxicity phenotypes were log-transformed to better approximate normal distributions. We tested for normality using the Shapiro-Wilk test and none of the drug phenotypes deviated significantly from normality (p>0.001). We selected this threshold because of the smaller sample size and also examined the frequency distribution to ensure that outliers were not substantially driving false positive associations. Protein expression and drug phenotypes were then tested for association with all markers genome-wide by linear regression implemented in Python and R using custom scripts. For each phenotype, we selected the most significantly associated SNV within each recombination window, defined by splitting the genome into 25,307 blocks flanked by >10 cM/Mb recombination rates estimated from HapMap.

### Enrichment analysis

For each drug, we generated 1,000 randomly selected sets of SNPs of the same size and matching the same MAF distribution as all SNPs significantly associated with that drug (dQTLs) at p<10^−3^ and examined the overlap of these dQTLs with pQTLs and eQTLs at p<10^−4^, as previously described [25]. We empirically determined the enrichment p-value by comparing the observed dQTL-pQTL or dQTL-eQTL SNP overlap to the null distribution. We also evaluated enrichment of dQTLs at p<10^−4^ for the SNP-transcript association to test the robustness of an enrichment result to the choice of p-value threshold. To investigate whether the observed enrichment of dQTLs to be pQTLs or eQTLs was driven by linkage disequilibrium, we performed an additional simulation analysis after selecting only the most significant dQTLs for each recombination block.

### siRNA nucleofection

LCLs were seeded at a density of 550,000 cells/mL 24 hours before nucleofection. Amaxa's Cell Line 96-well Nucleofector Kit SF (Lonza Inc, Basel, Switzerland) was used to perform the transfection. Cells were centrifuged at 90 g for 10 minutes at room temperature and resuspended at a concentration of 1,000,000 cells in 20 µL of SF/supplement solution (included in SF Kit Lonza Catalog #V4SC2096) and 2 µM final concentration of AllStars negative Control siRNA labeled with AlexaFluor488 (Qiagen Inc., Valencia, CA) or a pool of siRNA (Qiagen) (See Table S1). The cells were nucleofected using Amaxa's DN-100 program. Cells were allowed to rest for 10 minutes before the addition of pre-warmed (in 37° water bath for a minimum of 20 minutes) RPMI media and then another 5 minutes after the addition of warm RPMI media. Cells were then plated for protein measurements and drug treatments. Cells were harvested at 24 and 48 hours post-nucleofection for protein measurement. Drug treatment was done 18 hours following transfection for cell survival measurement and 24 hours after transfection for apoptosis measurement. Apoptosis was measured as described above, whereas cell survival was measured as described above for cisplatin and using Cell-Titer Glo (Promega) for paclitaxel. Each experiment was done twice, with two independent transfections.

### siRNA analyses

To assess the size and significance of the effect of siRNA knockdown on drug response (survival for cytotoxicity assay and caspase activity for apoptosis assay) we fit the following linear mixed effect model: 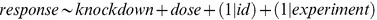, in which *knockdown* is 1 if the gene was knocked down and 0 if scrambled. Cell line id (denoted by *id*) and *experiment* were used as random effects to properly account for correlation between replicates. To increase precision, we pooled the data from all cell lines. The mixed effects model was fit using lme4 package in the R Statistical package (http://cran.r-project.org/). The goodness-of-fit of the model was assessed by examining the residuals. Normality of the residuals was assessed using the Shapiro-Wilk test in the R Statistical package. Log-transformation of the response variable was used to achieve approximate normality.

## Supporting Information

Figure S1 Cellular growth inhibition is inversely correlated with caspase 3/7 apoptosis measurements. In 68 unrelated YRI LCLs, both cisplatin (a) and paclitaxel (b) cytotoxic phenotypes were negatively correlated with apoptosis measurements. Paclitaxel's (c) correlation (r*^2^* = 0.35) was much stronger than cisplatin's (d) correlation (r*^2^* = 0.04).
(TIF)

Figure S2 Correlation of cellular phenotypes across thaw. We correlated the cellular phenotypes for cisplatin cytotoxicity (a) and apoptosis (b) and paclitaxel cytotoxicity (c) and apoptosis (d) using 63 cell lines for cytotoxicity and 21 for apoptosis. Both cytotoxicity phenotypes were correlated p<0.0001 with r^2^ of .28 (a) and .35 (c). Apoptosis phenotypes were correlated p<0.003 with r^2^ of .63 (b) and .38 (d).
(TIF)

Figure S3 Role of growth in proteins associated with chemotherapeutic phenotypes. SMC1A protein levels (a) are significantly correlated with intrinsic growth rate (p = 0.001) whereas ZNF569 (b) was not (p>0.05).
(TIF)

Figure S4 Minor allele frequency distribution comparing associated SNPs with all SNPs. SNPs associated with proteins' MAF distribution (middle) contained more common MAF variants than all SNPs tested genome-wide (left, two-sample Kolmogorov-Smirnov test p = 1, pQTL median MAF = 0.17 vs. genome-wide median MAF = 0.16). However, we appreciate that low MAF variants are statistically more prevalent in our dQTL associations (right) (C, K–S test p<0.05) but by not a large magnitude (dQTL median MAF = 0.15).
(TIF)

Table S1 siRNA used in functional experiments. The siRNAs that were purchased from Qiagen and pooled are listed for each gene indicated. The asterisk indicates that the siRNA was functionally validated to the target gene by Qiagen.
(DOC)

Table S2 Relationship between drug phenotypes and proteins. The mixed effect model (MEM) p-value and beta for all nominal (p<0.05) correlations between each phenotype (5 µM cisplatin apoptosis, 5 µM cisplatin cytotoxicity, 12.5 nM paclitaxel apoptosis, 12.5 nM paclitaxel cytotoxicity) and proteins are listed.
(XLSX)

Table S3 Overlap proteins implicated through apoptosis and cytotoxicity GWAS pQTLs. Genome-wide association results (p<10^−3^) on two cellular phenotypes (growth inhibition and apoptosis) for both cisplatin and paclitaxel were analyzed for pQTLs. All SNP that were pQTLs (p<10^−4^) had their target proteins evaluated for correlation with the drug phenotype (p≤0.05). For each drug, the target protein overlap between cytotoxicity and apoptosis is listed.
(DOC)

Table S4 SNPS correlated with drugs that are also pQTLs. Genome-wide association results (p<10^−3^) on two cellular phenotypes (growth inhibition and apoptosis) for both cisplatin and paclitaxel were analyzed for pQTLs (p<10^−4^) and are listed.
(XLSX)

Table S5 Antibodies used in this study. Information regarding the companies, antigens and gene names for the antibodies quantified in the 68 samples is provided.
(XLSX)

## References

[1] Wheeler HE, Dolan ME (2012) Lymphoblastoid cell lines in pharmacogenomic discovery and clinical translation. Pharmacogenomics 13: 55–70. 22176622 10.2217/pgs.11.121 PMC3292907

[2] Wheeler HE, Gamazon ER, Wing C, Njiaju UO, Njoku C, et al (2013) Integration of cell line and clinical trial genome-wide analyses supports a polygenic architecture of Paclitaxel-induced sensory peripheral neuropathy. Clin Cancer Res 19: 491–499. 23204130 10.1158/1078-0432.CCR-12-2618 PMC3549006

[3] Mitra AK, Crews KR, Pounds S, Cao X, Feldberg T, et al (2011) Genetic variants in cytosolic 5′-nucleotidase II are associated with its expression and cytarabine sensitivity in HapMap cell lines and in patients with acute myeloid leukemia. J Pharmacol Exp Ther 339: 9–23. 21712425 10.1124/jpet.111.182873 PMC3186292

[4] Ziliak D, O'Donnell PH, Im HK, Gamazon ER, Chen P, et al (2011) Germline polymorphisms discovered via a cell-based, genome-wide approach predict platinum response in head and neck cancers. Transl Res 157: 265–272. 21497773 10.1016/j.trsl.2011.01.005 PMC3079878

[5] Huang RS, Johnatty SE, Gamazon ER, Im HK, Ziliak D, et al (2011) Platinum sensitivity-related germline polymorphism discovered via a cell-based approach and analysis of its association with outcome in ovarian cancer patients. Clin Cancer Res 17: 5490–5500. 21705454 10.1158/1078-0432.CCR-11-0724 PMC3160494

[6] Niu N, Schaid DJ, Abo RP, Kalari K, Fridley BL, et al (2012) Genetic association with overall survival of taxane-treated lung cancer patients - a genome-wide association study in human lymphoblastoid cell lines followed by a clinical association study. BMC Cancer 12: 422. 23006423 10.1186/1471-2407-12-422 PMC3573965

[7] Brown CC, Havener TM, Medina MW, Auman JT, Mangravite LM, et al (2012) A genome-wide association analysis of temozolomide response using lymphoblastoid cell lines shows a clinically relevant association with MGMT. Pharmacogenet Genomics 22: 796–802. 23047291 10.1097/FPC.0b013e3283589c50 PMC3691078

[8] O'Donnell PH, Stark AL, Gamazon ER, Wheeler HE, McIlwee BE, et al (2012) Identification of novel germline polymorphisms governing capecitabine sensitivity. Cancer 118: 4063–4073. 22864933 10.1002/cncr.26737 PMC3413892

[9] Wheeler HE, Gamazon ER, Stark AL, O'Donnell PH, Gorsic LK, et al (2013) Genome-wide meta-analysis identifies variants associated with platinating agent susceptibility across populations. Pharmacogenomics J 13: 35–43. 21844884 10.1038/tpj.2011.38 PMC3370147

[10] Hartford CM, Duan S, Delaney SM, Mi S, Kistner EO, et al (2009) Population-specific genetic variants important in susceptibility to cytarabine arabinoside cytotoxicity. Blood 113: 2145–2153. 19109566 10.1182/blood-2008-05-154302 PMC2652364

[11] Huang RS, Duan S, Bleibel WK, Kistner EO, Zhang W, et al (2007) A genome-wide approach to identify genetic variants that contribute to etoposide-induced cytotoxicity. Proc Natl Acad Sci U S A 104: 9758–9763. 17537913 10.1073/pnas.0703736104 PMC1887589

[12] Duan S, Huang RS, Zhang W, Bleibel WK, Roe CA, et al (2008) Genetic architecture of transcript-level variation in humans. Am J Hum Genet 82: 1101–1113. 18439551 10.1016/j.ajhg.2008.03.006 PMC2651622

[13] Stranger BE, Montgomery SB, Dimas AS, Parts L, Stegle O, et al (2012) Patterns of cis regulatory variation in diverse human populations. PLoS Genet 8: e1002639. 22532805 10.1371/journal.pgen.1002639 PMC3330104

[14] Stranger BE, Nica AC, Forrest MS, Dimas A, Bird CP, et al (2007) Population genomics of human gene expression. Nat Genet 39: 1217–1224. 17873874 10.1038/ng2142 PMC2683249

[15] Veyrieras JB, Kudaravalli S, Kim SY, Dermitzakis ET, Gilad Y, et al (2008) High-resolution mapping of expression-QTLs yields insight into human gene regulation. PLoS Genet 4: e1000214. 18846210 10.1371/journal.pgen.1000214 PMC2556086

[16] Pickrell JK, Marioni JC, Pai AA, Degner JF, Engelhardt BE, et al (2010) Understanding mechanisms underlying human gene expression variation with RNA sequencing. Nature 464: 768–772. 20220758 10.1038/nature08872 PMC3089435

[17] Bell JT, Pai AA, Pickrell JK, Gaffney DJ, Pique-Regi R, et al (2011) DNA methylation patterns associate with genetic and gene expression variation in HapMap cell lines. Genome Biol 12: R10. 21251332 10.1186/gb-2011-12-1-r10 PMC3091299

[18] Pai AA, Cain CE, Mizrahi-Man O, De Leon S, Lewellen N, et al (2012) The contribution of RNA decay quantitative trait loci to inter-individual variation in steady-state gene expression levels. PLoS Genet 8: e1003000. 23071454 10.1371/journal.pgen.1003000 PMC3469421

[19] Degner JF, Pai AA, Pique-Regi R, Veyrieras JB, Gaffney DJ, et al (2012) DNase I sensitivity QTLs are a major determinant of human expression variation. Nature 482: 390–394. 22307276 10.1038/nature10808 PMC3501342

[20] Gamazon ER, Ziliak D, Im HK, LaCroix B, Park DS, et al (2012) Genetic architecture of microRNA expression: implications for the transcriptome and complex traits. Am J Hum Genet 90: 1046–1063. 22658545 10.1016/j.ajhg.2012.04.023 PMC3370272

[21] Ko DC, Gamazon ER, Shukla KP, Pfuetzner RA, Whittington D, et al (2012) Functional genetic screen of human diversity reveals that a methionine salvage enzyme regulates inflammatory cell death. Proc Natl Acad Sci U S A 109: E2343–2352. 22837397 10.1073/pnas.1206701109 PMC3435171

[22] Kato T, Hayashi-Takagi A, Toyota T, Yoshikawa T, Iwamoto K (2011) Gene expression analysis in lymphoblastoid cells as a potential biomarker of bipolar disorder. J Hum Genet 56: 779–783. 21866111 10.1038/jhg.2011.101

[23] Oved K, Morag A, Pasmanik-Chor M, Oron-Karni V, Shomron N, et al (2012) Genome-wide miRNA expression profiling of human lymphoblastoid cell lines identifies tentative SSRI antidepressant response biomarkers. Pharmacogenomics 13: 1129–1139. 22909203 10.2217/pgs.12.93

[24] Morag A, Oved K, Gurwitz D (2013) Sex differences in human lymphoblastoid cells sensitivities to antipsychotic drugs. J Mol Neurosci 49: 554–558. 22760742 10.1007/s12031-012-9852-z

[25] Gamazon ER, Huang RS, Cox NJ, Dolan ME (2010) Chemotherapeutic drug susceptibility associated SNPs are enriched in expression quantitative trait loci. Proc Natl Acad Sci U S A 107: 9287–9292. 20442332 10.1073/pnas.1001827107 PMC2889115

[26] Chen G, Gharib TG, Huang CC, Taylor JM, Misek DE, et al (2002) Discordant protein and mRNA expression in lung adenocarcinomas. Mol Cell Proteomics 1: 304–313. 12096112 10.1074/mcp.m200008-mcp200

[27] Gygi SP, Rochon Y, Franza BR, Aebersold R (1999) Correlation between protein and mRNA abundance in yeast. Mol Cell Biol 19: 1720–1730. 10022859 10.1128/mcb.19.3.1720 PMC83965

[28] Nishizuka S, Charboneau L, Young L, Major S, Reinhold WC, et al (2003) Proteomic profiling of the NCI-60 cancer cell lines using new high-density reverse-phase lysate microarrays. Proc Natl Acad Sci U S A 100: 14229–14234. 14623978 10.1073/pnas.2331323100 PMC283574

[29] Shankavaram UT, Reinhold WC, Nishizuka S, Major S, Morita D, et al (2007) Transcript and protein expression profiles of the NCI-60 cancer cell panel: an integromic microarray study. Mol Cancer Ther 6: 820–832. 17339364 10.1158/1535-7163.MCT-06-0650

[30] Vogel C, Abreu Rde S, Ko D, Le SY, Shapiro BA, et al (2010) Sequence signatures and mRNA concentration can explain two-thirds of protein abundance variation in a human cell line. Mol Syst Biol 6: 400. 20739923 10.1038/msb.2010.59 PMC2947365

[31] Mwanda WO, Orem J, Fu P, Banura C, Kakembo J, et al (2009) Dose-modified oral chemotherapy in the treatment of AIDS-related non-Hodgkin's lymphoma in East Africa. J Clin Oncol 27: 3480–3488. 19470940 10.1200/JCO.2008.18.7641 PMC2717754

[32] Ciaccio MF, Wagner JP, Chuu CP, Lauffenburger DA, Jones RB (2010) Systems analysis of EGF receptor signaling dynamics with microwestern arrays. Nat Methods 7: 148–155. 20101245 10.1038/nmeth.1418 PMC2881471

[33] Hause RJ, Kim HD, Leung KK, Jones RB (2011) Targeted protein-omic methods are bridging the gap between proteomic and hypothesis-driven protein analysis approaches. Expert Rev Proteomics 8: 565–575. 21999828 10.1586/epr.11.49 PMC3269123

[34] Zwelling LA, Kohn KW (1979) Mechanism of action of cis-dichlorodiammineplatinum(II). Cancer Treat Rep 63: 1439–1444. 387221

[35] Rowinsky EK, Cazenave LA, Donehower RC (1990) Taxol: a novel investigational antimicrotubule agent. J Natl Cancer Inst 82: 1247–1259. 1973737 10.1093/jnci/82.15.1247

[36] Pazdur R, Kudelka AP, Kavanagh JJ, Cohen PR, Raber MN (1993) The taxoids: paclitaxel (Taxol) and docetaxel (Taxotere). Cancer Treat Rev 19: 351–386. 8106152 10.1016/0305-7372(93)90010-o

[37] Ricci MS, Zong WX (2006) Chemotherapeutic approaches for targeting cell death pathways. Oncologist 11: 342–357. 16614230 10.1634/theoncologist.11-4-342 PMC3132471

[38] Im HK, Gamazon ER, Stark AL, Huang RS, Cox NJ, et al (2012) Mixed effects modeling of proliferation rates in cell-based models: consequence for pharmacogenomics and cancer. PLoS Genet 8: e1002525. 22346769 10.1371/journal.pgen.1002525 PMC3276560

[39] Zhang W, Duan S, Kistner EO, Bleibel WK, Huang RS, et al (2008) Evaluation of genetic variation contributing to differences in gene expression between populations. Am J Hum Genet 82: 631–640. 18313023 10.1016/j.ajhg.2007.12.015 PMC2427223

[40] Bergstrom Lind S, Artemenko KA, Elfineh L, Mayrhofer C, Zubarev RA, et al (2011) Toward a comprehensive characterization of the phosphotyrosine proteome. Cell Signal 23: 1387–1395. 21447384 10.1016/j.cellsig.2011.03.021

[41] Rojas AM, Sanchez-Pulido L, Futterer A, van Wely KH, Martinez AC, et al (2005) Death inducer obliterator protein 1 in the context of DNA regulation. Sequence analyses of distant homologues point to a novel functional role. FEBS J 272: 3505–3511. 16008551 10.1111/j.1742-4658.2005.04759.x

[42] Le XF, Bast RC Jr (2011) Src family kinases and paclitaxel sensitivity. Cancer Biol Ther 12: 260–269. 21646863 10.4161/cbt.12.4.16430 PMC3173729

[43] Chen T, Pengetnze Y, Taylor CC (2005) Src inhibition enhances paclitaxel cytotoxicity in ovarian cancer cells by caspase-9-independent activation of caspase-3. Mol Cancer Ther 4: 217–224. 15713893

[44] Van Waardenburg RC, Prins J, Meijer C, Uges DR, De Vries EG, et al (1996) Effects of c-myc oncogene modulation on drug resistance in human small cell lung carcinoma cell lines. Anticancer Res 16: 1963–1970. 8712728

[45] Walker TL, White JD, Esdale WJ, Burton MA, DeCruz EE (1996) Tumour cells surviving in vivo cisplatin chemotherapy display elevated c-myc expression. Br J Cancer 73: 610–614. 8605094 10.1038/bjc.1996.105 PMC2074343

[46] Gu F, Ma Y, Zhang Z, Zhao J, Kobayashi H, et al (2010) Expression of Stat3 and Notch1 is associated with cisplatin resistance in head and neck squamous cell carcinoma. Oncol Rep 23: 671–676. 20127005 10.3892/or_00000683

[47] Ikuta K, Takemura K, Kihara M, Nishimura M, Ueda N, et al (2005) Overexpression of constitutive signal transducer and activator of transcription 3 mRNA in cisplatin-resistant human non-small cell lung cancer cells. Oncol Rep 13: 217–222. 15643501

[48] Kato K, Nomoto M, Izumi H, Ise T, Nakano S, et al (2000) Structure and functional analysis of the human STAT3 gene promoter: alteration of chromatin structure as a possible mechanism for the upregulation in cisplatin-resistant cells. Biochim Biophys Acta 1493: 91–100. 10978511 10.1016/s0167-4781(00)00168-8

[49] Su WP, Cheng FY, Shieh DB, Yeh CS, Su WC (2012) PLGA nanoparticles codeliver paclitaxel and Stat3 siRNA to overcome cellular resistance in lung cancer cells. Int J Nanomedicine 7: 4269–4283. 22904633 10.2147/IJN.S33666 PMC3418083

[50] Walker SR, Chaudhury M, Frank DA (2011) STAT3 Inhibition by Microtubule-Targeted Drugs: Dual Molecular Effects of Chemotherapeutic Agents. Mol Cell Pharmacol 3: 13–19. 21949561 PMC3177107

[51] Wu H, Cao Y, Weng D, Xing H, Song X, et al (2008) Effect of tumor suppressor gene PTEN on the resistance to cisplatin in human ovarian cancer cell lines and related mechanisms. Cancer Lett 271: 260–271. 18657898 10.1016/j.canlet.2008.06.012

[52] Kim ST, Xu B, Kastan MB (2002) Involvement of the cohesin protein, Smc1, in Atm-dependent and independent responses to DNA damage. Genes Dev 16: 560–570. 11877376 10.1101/gad.970602 PMC155347

[53] Mannini L, Liu J, Krantz ID, Musio A (2010) Spectrum and consequences of SMC1A mutations: the unexpected involvement of a core component of cohesin in human disease. Hum Mutat 31: 5–10. 19842212 10.1002/humu.21129 PMC2797832

[54] Barber TD, McManus K, Yuen KW, Reis M, Parmigiani G, et al (2008) Chromatid cohesion defects may underlie chromosome instability in human colorectal cancers. Proc Natl Acad Sci U S A 105: 3443–3448. 18299561 10.1073/pnas.0712384105 PMC2265152

[55] Mannini L, Menga S, Tonelli A, Zanotti S, Bassi MT, et al (2012) SMC1A codon 496 mutations affect the cellular response to genotoxic treatments. Am J Med Genet A 158A: 224–228. 22140011 10.1002/ajmg.a.34384

[56] Chen S, Blank JL, Peters T, Liu XJ, Rappoli DM, et al (2010) Genome-wide siRNA screen for modulators of cell death induced by proteasome inhibitor bortezomib. Cancer Res 70: 4318–4326. 20460535 10.1158/0008-5472.CAN-09-4428

[57] Yamaguchi H, Durell SR, Chatterjee DK, Anderson CW, Appella E (2007) The Wip1 phosphatase PPM1D dephosphorylates SQ/TQ motifs in checkpoint substrates phosphorylated by PI3K-like kinases. Biochemistry 46: 12594–12603. 17939684 10.1021/bi701096s

[58] Huang X, Yuan W, Huang W, Bai Y, Deng Y, et al (2006) ZNF569, a novel KRAB-containing zinc finger protein, suppresses MAPK signaling pathway. Biochem Biophys Res Commun 346: 621–628. 16793018 10.1016/j.bbrc.2006.05.109

[59] Zhu C, Qi X, Chen Y, Sun B, Dai Y, et al (2011) PI3K/Akt and MAPK/ERK1/2 signaling pathways are involved in IGF-1-induced VEGF-C upregulation in breast cancer. J Cancer Res Clin Oncol 137: 1587–1594. 21904903 10.1007/s00432-011-1049-2 PMC11828124

[60] Slichenmyer WJ, Von Hoff DD (1991) Taxol: a new and effective anti-cancer drug. Anticancer Drugs 2: 519–530. 1687206

[61] Wen Y, Gamazon ER, Bleibel WK, Wing C, Mi S, et al (2012) An eQTL-based method identifies CTTN and ZMAT3 as pemetrexed susceptibility markers. Hum Mol Genet 21: 1470–1480. 22171072 10.1093/hmg/ddr583 PMC3298275

[62] Nicolae DL, Gamazon E, Zhang W, Duan S, Dolan ME, et al (2010) Trait-associated SNPs are more likely to be eQTLs: annotation to enhance discovery from GWAS. PLoS Genet 6: e1000888. 20369019 10.1371/journal.pgen.1000888 PMC2848547

[63] Gaffney DJ, Veyrieras JB, Degner JF, Pique-Regi R, Pai AA, et al (2012) Dissecting the regulatory architecture of gene expression QTLs. Genome Biol 13: R7. 22293038 10.1186/gb-2012-13-1-r7 PMC3334587

[64] Fu J, Wolfs MG, Deelen P, Westra HJ, Fehrmann RS, et al (2012) Unraveling the regulatory mechanisms underlying tissue-dependent genetic variation of gene expression. PLoS Genet 8: e1002431. 22275870 10.1371/journal.pgen.1002431 PMC3261927

[65] Huang RS, Duan S, Shukla SJ, Kistner EO, Clark TA, et al (2007) Identification of genetic variants contributing to cisplatin-induced cytotoxicity by use of a genomewide approach. Am J Hum Genet 81: 427–437. 17701890 10.1086/519850 PMC1950832

[66] Njiaju UO, Gamazon ER, Gorsic LK, Delaney SM, Wheeler HE, et al (2012) Whole-genome studies identify solute carrier transporters in cellular susceptibility to paclitaxel. Pharmacogenet Genomics 22: 498–507. 22437668 10.1097/FPC.0b013e328352f436 PMC3376193

