# Supplementary figures and images for: Protein Quantitative Trait Loci Identify Novel Candidates Modulating Cellular Response to Chemotherapy
Source: PLoS Genet. 2014 Apr 3;10(4):e1004192. doi: 10.1371/journal.pgen.1004192 (PMC3974641; doi:10.1371/journal.pgen.1004192)

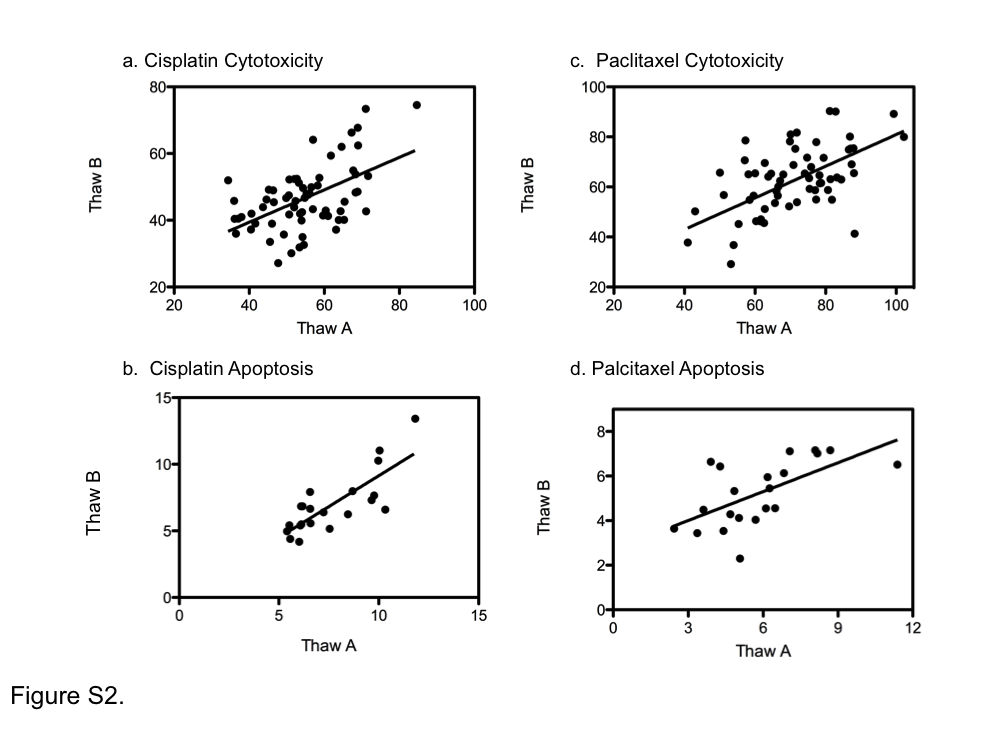

Supplement: Figure S2 — Correlation of cellular phenotypes across thaw. We correlated the cellular phenotypes for cisplatin cytotoxicity (a) and apoptosis (b) and paclitaxel cytotoxicity (c) and apoptosis (d) using 63 cell lines for cytotoxicity and 21 for apoptosis. Both cytotoxicity phenotypes were correlated p<0.0001 with r2 of .28 (a) and .35 (c). Apoptosis phenotypes were correlated p<0.003 with r2 of .63 (b) and .38 (d). (TIF) [file pgen.1004192.s002.tif]
