# Supplementary material for: Protein Quantitative Trait Loci Identify Novel Candidates Modulating Cellular Response to Chemotherapy
Source: PLoS Genet. 2014 Apr 3;10(4):e1004192. doi: 10.1371/journal.pgen.1004192 (PMC3974641; doi:10.1371/journal.pgen.1004192)
Supplement: Table S1 — siRNA used in functional experiments. The siRNAs that were purchased from Qiagen and pooled are listed for each gene indicated. The asterisk indicates that the siRNA was functionally validated to the target gene by Qiagen. (DOC) [file pgen.1004192.s005.doc]

Table S1. siRNA used in functional experiments

| **FlexiTube GeneSolution GS11083** | **FlexiTube GeneSolution GS8243** | **FlexiTube GeneSolution GS148266** |
| --- | --- | --- |
| DIDO1  Entrez gene ID: 11083 | SMC1A  Entrez gene ID: 8243 | ZNF569  Entrez gene ID: 148266 |
| SI03111185 | SI00087241 | SI04372963 |
| SI00132552 | SI00087255 | SI03156503 |
| SI00132559 | SI00087248 | SI04202639 |
| SI03080042 | SI02655219* | SI04219628 |
