# Supplementary material for: Protein Quantitative Trait Loci Identify Novel Candidates Modulating Cellular Response to Chemotherapy
Source: PLoS Genet. 2014 Apr 3;10(4):e1004192. doi: 10.1371/journal.pgen.1004192 (PMC3974641; doi:10.1371/journal.pgen.1004192)
Supplement: Table S3 — Overlap proteins implicated through apoptosis and cytotoxicity GWAS pQTLs. Genome-wide association results (p<10−3) on two cellular phenotypes (growth inhibition and apoptosis) for both cisplatin and paclitaxel were analyzed for pQTLs. All SNP that were pQTLs (p<10−4) had their target proteins evaluated for correlation with the drug phenotype (p≤0.05). For each drug, the target protein overlap between cytotoxicity and apoptosis is listed. (DOC) [file pgen.1004192.s007.doc]

Table S3. Overlap Proteins implicated through apoptosis and cytotoxicity GWAS pQTLs.

| Drug | Protein | Gene | Apoptosis P-Value Mixed Effect | Apoptosis Q-Value Mixed Effect | Apoptosis Beta Mixed Effect | Cytotoxicity P-Value Mixed Effect | Cytotoxicity Q-Value Mixed Effect | Cytotoxicity Beta  Mixed Effect |
| --- | --- | --- | --- | --- | --- | --- | --- | --- |
| Cisplatin | RAXL1 | RAX2 | 0.034 | 0.901 | -0.37 | 0.05 | 0.866 | 0.596 |
|  | SMC1A | SMC1A | 0.052 | 0.326 | -0.256 | 0.005 | 0.985 | 0.67 |
|  | Src.A070 | SRC | 0.012 | 0.995 | 0.4 | 0.017 | 0.813 | -0.755 |
|  | TFAP2A | TFAP2A | 0.006 | 0.724 | -0.49 | 0.012 | 0.758 | 0.775 |
|  | ZNF645 | ZNF645 | 0.035 | 0.763 | 0.308 | 0.048 | 0.612 | -0.505 |
| Paclitaxel | AFF2 (50kDa) | AFF2 | 0.014 | 0.995 | -0.367 | 0.049 | 0.995 | 0.705 |
|  | DIDO1 | DIDO1 | 0.015 | 0.966 | 0.354 | 0.006 | 0.883 | -0.925 |
|  | ENO1 | ENO1 | 0.00001 | 0.801 | -0.472 | 0.005 | 0.92 | 0.83 |
|  | FAK | PTK2 | 0.009 | 0.861 | 0.403 | 0.005 | 0.924 | -0.997 |
|  | GTF2F2 | GTF2F2 | 0.003 | 0.864 | 0.407 | 0.0003 | 0.864 | -1.154 |
|  | HMX1 | HMX1 | 0.047 | 0.728 | .285 | .04 | 0.612 | -0.684 |
|  | JunB | JUNB | 0.029 | 0.993 | -0.373 | 0.022 | 0.875 | 0.946 |
|  | IRF5 | IRF5 | 0.013 | 0.920 | -.034 | 0.0005 | .920 | 1.08 |
|  | p-S6.  ribosomal.protein.(S240/244) | RPS6 | 0.005 | 0.997 | -0.389 | 0.005 | 0.997 | 0.903 |
|  |  |  |  |  |  |  |  |  |
|  | P70.S6. | RPS6KB1 | 0.017 | 0.836 | 0.432 | 0.02 | 0.704 | -1.031 |
|  | Kinase |  |  |  |  |  |  |  |
|  | PCGF3 | PCGF3 | 0.035 | 0.744 | 0.282 | 0.038 | 0.894 | -0.641 |
|  | SCRT1 | SCRT1 | 0.007 | 0.644 | -0.347 | 0.016 | 0.878 | 0.717 |
|  | SP1 | SP1 | 0.006 | 0.92 | 0.409 | 0.035 | 0.948 | -0.756 |
|  | Src.A070 | SRC | 0.002 | 0.894 | 0.414 | 0.009 | 0.894 | -0.834 |
|  | Src.A071 | SRC | 0.014 | 0.871 | 0.408 | 0.014 | 0.894 | -0.956 |
|  | STAT3A (95kDa) | STAT3 | 0.001 | 0.895 | -0.416 | 0.00001 | 0.635 | 1.23 |
|  | STAT3B (85kDa) | STAT3 | 0.034 | 0.901 | -0.271 | 0.001 | 0.895 | 0.884 |
|  | TAF15 | TAF15 | 0.035 | 0.986 | -0.325 | 0.007 | 0.726 | 0.962 |
|  | ZNF266 (75-100 kDa) | ZNF266 | 0.004 | 0.912 | 0.311 | 0.0003 | 0.912 | -0.894 |
|  | ZNF267 | ZNF267 | 0.03 | 0.8 | -0.28 | 0.001 | 0.926 | 1.026 |
|  | ZNF569 | ZNF569 | 0.004 | 0.71 | 0.396 | 0.043 | 0.941 | -0.693 |
